# Supplementary material for: Adapting a guided low-intensity behavioural activation intervention for people with dementia and depression in the Swedish healthcare context (INVOLVERA): a study protocol using codesign and participatory action research
Source: BMJ Open. 2021 Jul 16;11(7):e048097. doi: 10.1136/bmjopen-2020-048097 (PMC8287613; doi:10.1136/bmjopen-2020-048097)
Supplement: Author's manuscript [file bmjopen-2020-048097.draft_revisions.pdf]

BMJ Open is committed to open peer review. As part of this commitment we make the peer review history of every article we publish publicly available.

When an article is published we post the peer reviewers' comments and the authors' responses online. We also post the versions of the paper that were used during peer review. These are the versions that the peer review comments apply to.

The versions of the paper that follow are the versions that were submitted during the peer review process. They are not the versions of record or the final published versions. They should not be cited or distributed as the published version of this manuscript.

BMJ Open is an open access journal and the full, final, typeset and author-corrected version of record of the manuscript is available on our site with no access controls, subscription charges or pay-per-view fees (http://bmjopen.bmj.com).

If you have any questions on BMJ Open's open peer review process please email info.bmjopen@bmj.com

BMJ Open

# Adapting a guided low-intensity behavioural activation intervention for people with dementia and depression in the Swedish healthcare context (INVOLVERA): a study protocol utilising co-design and participatory action research

| | |
|---|---|
| Journal: | *BMJ Open* |
| Manuscript ID | bmjopen-2020-048097 |
| Article Type: | Protocol |
| Date Submitted by the Author: | 16-Dec-2020 |
| Complete List of Authors: | Svedin, Frida; Uppsala University, Clinical Psychology in Healthcare, Department of Women's and Children's Health<br>Brantnell, Anders; Uppsala University, Clinical Psychology in Healthcare, Department of Children's and Women's Health; Uppsala University, Division of Industrial Engineering and Management, Department of Civil and Industrial Engineering<br>Farrand, Paul; University of Exeter, Clinical Education Development and Research (CEDAR)<br>Blomberg, Oscar; Uppsala University, Clinical Psychology in Healthcare, Department of Women's and Children's Health<br>Coumoundouros, Chelsea; Uppsala University, Clinical Psychology in Healthcare, Department of Women's and Children's Health<br>von Essen, Louise; Uppsala University, Clinical Psychology in Healthcare, Department of Women’s and Children’s Health<br>Åberg, Anna ; Uppsala University, Geriatrics, Department of Public Health and Caring Sciences ; Dalarna University, Medical Science, School of Education, Health and Social Studies<br>Woodford, Joanne; Uppsala University, Clinical Psychology in Healthcare, Department of Women´s and Children´s Health |
| Keywords: | Dementia < NEUROLOGY, MENTAL HEALTH, GERIATRIC MEDICINE, Depression & mood disorders < PSYCHIATRY |
| | |

SCHOLARONE™
Manuscripts

*I, the Submitting Author has the right to grant and does grant on behalf of all authors of the Work (as defined in the below author licence), an exclusive licence and/or a non-exclusive licence for contributions from authors who are: i) UK Crown employees; ii) where BMJ has agreed a CC-BY licence shall apply, and/or iii) in accordance with the terms applicable for US Federal Government officers or employees acting as part of their official duties; on a worldwide, perpetual, irrevocable, royalty-free basis to BMJ Publishing Group Ltd ("BMJ") its licensees and where the relevant Journal is co-owned by BMJ to the co-owners of the Journal, to publish the Work in this journal and any other BMJ products and to exploit all rights, as set out in our licence.*

*The Submitting Author accepts and understands that any supply made under these terms is made by BMJ to the Submitting Author unless you are acting as an employee on behalf of your employer or a postgraduate student of an affiliated institution which is paying any applicable article publishing charge ("APC") for Open Access articles. Where the Submitting Author wishes to make the Work available on an Open Access basis (and intends to pay the relevant APC), the terms of reuse of such Open Access shall be governed by a Creative Commons licence – details of these licences and which Creative Commons licence will apply to this Work are set out in our licence referred to above.*

*Other than as permitted in any relevant BMJ Author's Self Archiving Policies, I confirm this Work has not been accepted for publication elsewhere, is not being considered for publication elsewhere and does not duplicate material already published. I confirm all authors consent to publication of this Work and authorise the granting of this licence.*

For peer review only

# Adapting a guided low-intensity behavioural activation intervention for people with dementia and depression in the Swedish healthcare context (INVOLVERA): a study protocol utilising co-design and participatory action research

Frida SVEDIN[1] (frida.svedin@kbh.uu.se), Anders BRANTNELL[1,2] (anders.brantnell@kbh.uu.se), Paul FARRAND[3] (p.a.farrand@exeter.ac.uk), Oscar BLOMBERG[1] (oscar.blomberg@kbh.uu.se), Chelsea COUMOUNDOUROS[1] (chelsea.coumoundouros@kbh.uu.se), Louise VON ESSEN[1] (louise-von.essen@kbh.uu.se), Anna Cristina ÅBERG[4,5] (anna.cristina.aberg@pubcare.uu.se), Joanne WOODFORD[1] (joanne.woodford@kbh.uu.se)

**Corresponding author**

Joanne Woodford, Clinical Psychology in Healthcare, Department of Women's and Children's Health, Uppsala University, 751 85 Uppsala, Sweden. E-mail: joanne.woodford@kbh.uu.se. Telephone: 0046 729 999 211

**Affiliations**

[1]Clinical Psychology in Healthcare, Department of Women's and Children's Health, Uppsala University, 751 85 Uppsala, Sweden.

[2]Division of Industrial Engineering and Management, Department of Civil and Industrial Engineering, Uppsala University, 751 21 Uppsala, Sweden

[3]Clinical Psychology, Education, Development and Research (CEDAR), Psychology, University of Exeter, Perry Road, Devon, EX4 4QG, United Kingdom

[4]Geriatrics, Department of Public Health and Caring Sciences, Uppsala University, 751 85 Uppsala, Sweden

For peer review only

[5]Medical Science, School of Education, Health and Social Studies, Dalarna University, 791 88 Falun, Sweden

**Word count: 3384 (excluding: title page, abstract, acknowledgements, contributions, and references)**

For peer review only

**ABSTRACT**

**Introduction:** Dementia is a worldwide health concern with incident rates continuing to increase. Whilst depression prevalence is high in people with dementia, and psychological interventions such as cognitive behavioral therapy are effective, access to psychological interventions remains limited. Reliance on traditional cognitive behavioral therapy for people with dementia and depression may present difficulties given it is a complex psychological approach, costly to deliver, and professional training time is lengthy. An alternative approach is behavioural activation, a simpler psychological intervention for depression. The present study seeks to work with people with dementia, informal caregivers, community stakeholders, and healthcare professionals, to adapt a guided low-intensity behavioural activation intervention for people with dementia and depression whilst maximising implementation potential within the Swedish healthcare context.

**Methods and analysis:** A mixed-methods study utilising Co-Design, Participatory Action Research (PAR) and Normalization Process Theory (NPT) to facilitate the cultural relevance, appropriateness, and implementation potential of the intervention. The study will consist of four iterative PAR phases, using focus groups with key community stakeholders and healthcare professionals, and semi-structured interviews with people with dementia and informal caregivers. A content analysis approach will be adopted to analyse the transcribed focus group recordings and semi-structured interviews.

**Ethics and dissemination:** The study will be conducted in accordance with the Helsinki Declaration and data handled according to General Data Protection Regulation. Written informed consent will be obtain from all study participants. In accordance with Swedish Health and Medical Services Act, capacity to consent will be examined by a member from the research team. Ethical approval has been obtained from the Swedish Ethical Review Authority (Dnr: 2020-05542). Findings will be published in an open access peer-reviewed journal, presented at academic conferences, and disseminated amongst lay and healthcare professional audiences.

For peer review only

## STRENGTHS AND LIMITATIONS OF THIS STUDY

- To the best of our knowledge, this is the first study in Sweden integrating Co-Design, Participatory Action Research, and Normalization Process Theory to develop a psychological intervention for depression in people with dementia and depression.
- Including key community stakeholders and healthcare professionals can help ensure the intervention is truly clinic- and patient-centered due to their 'real-world' knowledge and ability to provide useful information concerning how to efficiently implement the intervention.
- Utilization of implementation theory i.e. Normalization Process Theory should improve implementation potential and potentially optimize effectiveness.
- Co-Design and Participatory Action Research are time and resource consuming approaches.
- With limited resourses, this study will not be able to include ethnic minority groups due to the increasing costs for e.g. recruitment, informed consent and need for translated materials.

**KEY WORDS**

Dementia, depression, Co-Design, Participatory Action Research, Normalization Process Theory, cultural adaptation

For peer review only

# INTRODUCTION

## Background

Dementia is a worldwide health concern, with the number of people living with dementia set to rise from approximately 44 million in 2016[1] to over 140 million by 2050.[2] In Sweden, over 140,000 people are living with dementia,[1] with around 25,000 incident cases each year.[3] Further, the societal cost of dementia in Sweden is high and was estimated at nearly 63 billion SEK during 2012.[3] Due to both prevalence rates and the significant burden placed on individuals and wider society, dementia represents a challenge for global healthcare policy,[4] especially given the lack of curative or preventative medical interventions.[5] Indeed, dementia is expected to represent the largest increase in serious health-related suffering and burden globally between 2016-2060,[6] and is associated with increased mortality,[7] poor quality of life,[8] and poor functional outcomes.[9] Further contributing to health-related suffering are elevated symptoms of depression, with prevalence rates estimated to be between 30-87%.[10-13] Depression in people with dementia is associated with increased mortality,[14] poor quality of life,[10] sleep difficulties,[15] and reduction in activities of daily living.[10] Despite depression being highly prevalent, and evidence-based psychological interventions such as cognitive behavioral therapy (CBT) being effective for people with mild-to-moderate dementia,[16] access remains limited.[17] Lack of access to evidence-based psychological interventions is of particular importance since pharmacological approaches for depression show poor efficacy and negative side effects in people with dementia.[18-19]

However, this psychological "treatment gap" is not unique to a dementia population, with only 30% of people across Europe with mental health difficulties accessing support.[20] To address the psychological treatment gap, there have been global efforts to increase access to psychological interventions through the provision of Low-Intensity CBT (LI-CBT).[21-22] LI-CBT can be characterised as a single-strand approach, adopting a single CBT technique to target specific common mental health difficulties where an evidence base has been demonstrated.[23] Further, LI- CBT is delivered in a self-help format via a health technology (e.g., written workbooks, audio books, internet-administered, smartphone applications),[21] with the provision of face-to-face, telephone, or email guidance associated with larger effect sizes.[23] For example, across England, guidance to LI-CBT is provided by a psychological practitioner workforce [23] with graduate or postgraduate level training in the competencies required to support LI-CBT.[24]

For peer review only

LI-CBT may represent a solution to improve access for people with dementia and depression. One evidence based LI-CBT technique for depression is simple behavioural activation (BA). The simple BA model seeks to target behavioural avoidance (e.g., disengagement from pleasurable, routine, and necessary activities) [25], a common symptom of both depression [26] and dementia,[27] by increasing engagement in activities. As such, BA may also help overcome sedentary behaviours common in older adults [28], which may lead to improved functioning in people with dementia (e.g., engagement in, and ability to perform, activities of daily life). Further, the simple BA model is a straightforward psychological approach [29] and may be easier for people with dementia to understand than more complex CBT techniques. In addition to showing promise for people with dementia and depression, BA may also benefit informal caregivers [30] given improved functioning in the person with dementia is associated with decreases in caregiver burden.[30] Further, informal caregivers may engage more in activities with the person with dementia, possibly improving caregivers own mental health.[31] This is of particular importance given global and social care priorities to support people with dementia to remain at home for as long as possible [32] have resulted in an increased reliance on support from informal caregivers.[33]

Given the promise of BA, a research programme, informed by Phase I (development) of the Medical Research Council (MRC) complex interventions framework,[34-35] has been undertaken in England [29]. However, before applying the intervention in Sweden, there is a need to adapt the intervention to fit into the Swedish cultural [36] and healthcare context [37] whilst maintaining fidelity to the intervention.[38] For example, at present there is no psychological practitioner workforce in Sweden specifically trained in supporting LI-CBT.[39] In addition, it is increasingly recognised that there are multiple facilitators and barriers to the implementation of evidence-based interventions, e.g., problems with adaptation, fidelity, and delivery capability.[40] Normalisation Process Theory (NPT) provides a framework for understanding key mechanisms that facilitate or hinder the implementation of complex intervention.[41-43] NPT proposes four key constructs that enable the normalisation of complex intervention by stakeholders: (1) coherence (individual and collective sense-making); (2) cognitive participation (engagement to build and sustain practice); (3) collective action (enact practices); and (4) reflective monitoring (appraisal).[42,44] Traditionally, NPT has been used to analyse the implementation of

For peer review only

complex interventions.[45] However, more recently NPT has been used inform the development of complex interventions to enhance implementation potential.[46-47]

## Aim and objectives

The overall aim is to adapt the guided low-intensity BA intervention developed in England for people with dementia and depression and their informal caregivers, and enhance implementation potential for the Swedish cultural and healthcare context. Objectives are to:

1. Develop an understanding of the existing community and healthcare support context for people with dementia and their informal caregivers;
2. Identify barriers and facilitators to intervention uptake;
3. Identify feasible, acceptable, and implementable evidence-based methods of intervention delivery informed by NPT;
4. Adapt the guided low-intensity BA intervention to ensure cultural appropriateness, relevancy, and acceptability to people with dementia and their informal caregivers.

# METHODS AND ANALYSIS

## Study design

A mixed-methods study utilising Co-Design and Participatory Action Research (PAR)[48-49], placing key stakeholders (e.g., people with dementia, informal caregivers, healthcare professionals, and community stakeholders) at the centre of the research process.[48]

## Setting

In Sweden, counties are predominately responsible for general healthcare [50], with municipalities responsible for general and specialised care and services in the home, nursing homes, day care, respite care, and support for informal caregivers. [51] This study will seek to involve key stakeholders from both general and specialised health and social care services. The study will take place in Uppsala County, a county with both rural and urban areas. Uppsala County has approximately 382 000 residents [52] with more than 5 000 people estimated having dementia.[53]

## Study participants

Healthcare professionals and community stakeholders

For peer review only

Healthcare professionals (n=8) in the Uppsala area, e.g., primary care and hospital based physicians involved in dementia diagnosis, dementia care consultants, memory service nurses, nursing assistants working with people with dementia in day care centre settings and clinical psychologists will be recruited. Key community stakeholders (n=6) from relevant non-profit organizations will also be recruited, e.g., from Dementia League, Alzheimer Sweden and Red Cross.

People with dementia and informal caregivers

The following inclusion criteria will be applied for people with dementia (n=10):

1. Have a probable diagnosis of dementia (any type);
2. Live at home;
3. Able to and have the capacity to provide informed consent;
4. Able to speak and understand Swedish.

People with dementia will be excluded if they have:

1. A self-reported diagnosis of a severe and enduring mental health difficulty (e.g. psychosis, type I or II bipolar disorder and personality disorder);
2. A visual or auditory impairment that would hinder their ability to participate in semi-structured interviews and/or give feedback on the intervention;
3. A self-reported misuse of alcohol or prescription/non-prescription drugs so severe it interferes with their ability to perform normal activities in daily life.

The following inclusion criteria will be applied for informal caregivers (n=10):

1. Self-identified informal caregiver of a person with dementia with whom they have regular contact (at least weekly);
2. 18 years of age or older;
3. Able to speak, understand and write in Swedish.

Informal caregivers will be excluded if they have:

1. A self-reported diagnosis of a severe and enduring mental health difficulty (e.g. psychosis, type I or II bipolar disorder and personality disorder);
2. A visual or auditory impairment that would hinder their ability to participate in semi-structured interviews and/or give feedback on the intervention;

For peer review only

3. A self-reported misuse of alcohol or prescription/non-prescription drugs so severe it interferes with their ability to perform normal activities in daily life.

**Recruitment**

Healthcare professionals and community stakeholders

Healthcare professionals and key community stakeholders will be identified via in person-networks, social media, posters on notice boards and printed flyers.[54] A member of the research team will speak to interested stakeholders over the telephone to provide more information alongside a study invitation pack by post or email containing a: (1) study invitation letter; (2) study information sheet; (3) reply slip; (4) reasons for non-participation questionnaire; and (5) stamped addressed envelope.

People with dementia and informal caregivers

A multifaceted approach to recruitment will be adopted across four settings:

*Academic healthcare centres*

A research nurse will conduct patient record searches to identify people with dementia against the inclusion/exclusion criteria. When identified, a research nurse will make contact and ask verbal permission, face-to-face or via telephone, for a member of the research team to contact them. If permission is granted, a member of the research team will contact the person with dementia over the telephone to provide more study information and send a study information pack by post to interested potential participants. Study information packs include a: (1) study invitation letter; (2) study information sheet; (3) reply slip; (4) reasons for non-participation questionnaire, and (5) a stamped addressed envelope. The study information pack will also include an envelope containing a separate study invitation pack to be passed to an informal caregiver.

*Memory clinic*

A physician will provide people with dementia and/or informal caregivers with brief verbal study information alongside a study information pack (as above) during diagnosis meetings or follow-up visits (≤12 months since diagnosis).

*Day care centres*

For peer review only - http://bmjopen.bmj.com/site/about/guidelines.xhtml

Day care centre staff will provide informal caregivers with brief verbal study information and a study invitation pack (as above).

*Dementia care consultants*

Dementia care consultants will provide people with dementia and/or informal caregivers with brief verbal study information alongside a study invitation pack (as above).

**Informed consent and eligibility screening**

Healthcare professionals and community stakeholders

Healthcare professionals and community stakeholders respond to the study invitation by returning a reply slip, telephoning, or e-mailing the research team. A member of the research team will speak to those expressing interest in participation. During this phone call, interested stakeholders will have the opportunity to ask further questions about the study. Written informed consent will be obtained either face-to-face or via the post, or alternatively, verbal recorded consent will be obtained over the telephone, or via secure videoconferencing system. After provision of informed consent, background and socio-demographic characteristics— age, gender, profession, length of time in profession, professional qualifications, and length of time working with dementia – will be collected.

People with dementia and informal caregivers

People with dementia and informal caregivers can respond to the research team by returning a reply slip, telephoning, or e-mailing. Those expressing interest in participation will be contacted by a member of the research team who will arrange a meeting (either face-to-face, over the telephone, or via secure videoconferencing system) to gain informed consent and undertake an eligibility screen. In accordance with the Swedish Health and Medical Services Act,[55] capacity to consent in people with dementia will be assessed by a member of the research team, with appropriate knowledge and training, at the meeting. People with dementia will be provided with appropriate support to maximise ability to provide informed consent,[56] for example the provision of dementia-friendly written information.[57] Written informed consent will be obtained for people with dementia and informal caregivers met face-to-face and verbal recorded informed consent will be obtained by those met over telephone or secure videoconferencing.

For peer review only

After provision of informed consent, eligibility screening, background and socio-demographic characteristics will be collected. Given people with dementia may find it difficult to provide this data, an informal caregiver will be asked to attend the meeting and provide proxy data if needed.

**Reasons for non-participation**

Study information packs for will include an anonymous reasons for refusal of participation form for those who decline participation. The reasons for refusal of participation consists of a short questionnaire including possible causes for refusal informed by previous research[58-59] and an open-ended question. Reasons for refusal of participation will provide important information regarding recruitment feasibility and the acceptability of the proposed intervention.[59-60]

**PAR Phases**

PAR Phase I

Two focus group meetings (90-120 minutes) will be held with healthcare professionals and key community stakeholders, with semi-structured interviews offered to stakeholders unable to attend a focus group. Prior to the focus group/interview, a written prototype of the BA intervention will be provided. A topic guide, informed by NPT [42,44] will consist of open-ended questions examining:

1. Perceived purpose of the intervention;
2. First impressions of the intervention;
3. Potential impact of the intervention on people with dementia and informal caregivers;
4. Perceived differences and similarities to existing support available for people with dementia and informal caregivers;
5. Barriers and facilitators to people with dementia and informal caregivers using the intervention;
6. Potential professional/non-professional group to support and guide people with dementia and informal caregivers using the intervention (e.g., dementia care consultants, nurses, psychologists, volunteers);
7. Preferred intervention delivery setting (e.g., home, primary healthcare centre, memory clinic, day care);

For peer review only

8. Type of support and guidance (e.g., telephone, face-to-face, online) needed to facilitate people with dementia and informal caregivers using the intervention; and
9. Anticipated barriers and facilitators to the potential professional/non-professional group implementing the intervention (e.g., resources, training, managerial support, change agents).

Focus groups/interviews may be held face-to-face, via videoconference, or over the telephone, dependent on preference, and will be audio recorded. The research team will keep a reflective journal, logging the progresses, obstacles, and successes of the research process.[61]

PAR Phase II

Individual semi-structured interviews will be held with people with dementia and informal caregivers. Prior to the interview, the written prototype of the BA intervention will be provided. A topic guide, informed by NPT [42, 44] will examine similar topics to those examined in PAR Phase I. Additional focus will be placed on the perceived relevance of the intervention content and language and ways to enhance relevancy, cultural appropriateness, and acceptability. Interviews (45-60 minutes) will be held face-to-face (in a convenient location for the person with dementia and informal caregiver, e.g., at their home or other familiar community location chosen by the participant), or via videoconference/telephone.

PAR Phase III

Based on PAR Phase I and II results, a new version of the intervention and proposed model of delivery (e.g., professional/non-professional group to support and guide people with dementia and informal caregivers using the intervention, intervention delivery and type of support and guidance) will be developed. Two focus group meetings will be held with healthcare professionals and key community stakeholders from PAR Phase I, with semi-structured interviews offered to those unable to attend. A topic guide, similar to PAR Phase I will guide focus groups/interviews and examine the implementation potential of the new version of the intervention in accordance with NPT. At the end of the focus groups/interviews the Swedish version of the Normalization Process Theory Measure (S-NoMAD) [62] will be completed to examine implementation potential.

PAR Phase IV

For peer review only

A second round of semi-structured interviews with the same people with dementia and informal caregivers who participated in PAR Phase II will be conducted. A new version of the intervention and proposed delivery model established in PAR Phases I-III will be presented. A topic guide, similar to PAR Phase II, will guide interviews and examine the implementation potential of the new version of the intervention in accordance with NPT.

## Data analysis

Focus groups/interviews will be transcribed and uploaded into NVivo version 12.0 to support data analysis. Data analysis will be iterative, e.g., will be conducted after each PAR Phase to inform the subsequent phase. A content analysis approach will be adopted to analyse transcribed recordings.[63] NPT constructs will inform deductive coding, however inductive coding will be applied to relevant data unrelated to NPT constructs. S-NoMAD results will be analysed descriptively [62] in SPSS version 25 and used to triangulate qualitative findings.

## Trustworthiness

Trustworthiness will be established via the use of a trustworthiness checklist developed for content analysis.[64] To ensure study credibility, peer examination, field journals, and member checking will be conducted. Dense description and presenting demographic data will be incorporated to ensure transferability.[65] Two members of the research team will independently identify codes and themes,[65] and thereafter compare their findings. Codes and themes will also be discussed by the wider research team and Patient and Public Involvement group (see section on Patient and Public Involvement).

## Reporting

Qualitative results will be reported through the Standards for Reporting Qualitative Research (SRQR).[66] Intervention content will be reported in accordance with the Template for Intervention Description and Replication (TIDieR).[67] Patient and Public involvement will follow Guidance for Reporting Patient and Public Involvement (GRIPP2).[68]

## Patient and public involvement

A Patient and Public Involvement (PPI) group consisting of people with dementia (n=3) and informal caregivers (n=3) will be established to work alongside the research team as active research partners throughout the research process. The PPI group will be recruited via

For peer review only

advertisement through existing networks, relevant online/social media groups, and relevant local healthcare settings. The PPI group will meet together with the research team once every two-three months, with meetings held face-to-face, via video conference and/or telephone depending on group members' preferences. The PPI group will assist with research activities, such as advising on recruitment and research procedures, co-development of the intervention materials, interpretation of study results, and dissemination. A member of the research team will log all PPI activities in an impact log, which will be distributed to PPI group members immediately after each meeting to comment on accuracy.[69]

## DISCUSSION

At present, the psychological support needs of people with dementia and depression, living at home are not met by the Swedish healthcare system.[70] The proposed intervention has the potential to meet this need and support global priorities to support people with dementia to 'live well' with dementia and promote 'healthy ageing.' The intervention has further potential to reduce caregiver burden by providing effective strategies for informal caregivers to support the people with dementia.[31] The planned study is designed to improve the implementation potential of the intervention and will begin to explore intervention feasibility and acceptability. Results will be used to inform the design of a phase II feasibility following the MRC framework for the development and evaluation of complex interventions.[35]

## ETHICS AND DISSEMINATION

The study will be conducted in accordance with Helsinki Declaration.[71] All data will be handled according to General Data Protection Regulation (GDPR).[72] Informed consent will be obtained from all study participants [73] and capacity to consent in people with dementia will be established in accordance with Swedish Health and Medical Services Act.[55] Capacity to consent will be examined by a member from the research team. The study is approved by the Swedish Ethical Review Authority (Dnr: 2020-05542). Findings will be published in an open access peer-reviewed journal. We will aim to present results at scientific conferences as well as publications for public and professional audiences. The research team and PPI group will also promote results via social media.

For peer review only

## AUTHORS' CONTRIBUTIONS

FS: Methodology, resources, writing – original draft, project administration; AB: Writing – original draft, project administration, supervision; PF: conceptualisation, writing – review and editing; ACÅ: methodology, writing – review and editing; OB: writing – review and editing, project administration; CC: writing – review and editing, LvE: Writing – review and editing; JW: Conceptualisation, methodology, writing - original draft, supervision, project administration, funding acquisition.

## FUNDING

This work is funded by the Swedish Research Council (Dnr: 2018-02691). Funders were not involved in the creation, development or publication of this protocol. Funders will not be involved in the conduct, analysis or reporting of the resulting study.

## COMPETING INTERESTS

The authors declare they have no conflict of interests.

## COPYRIGHT AND AUTHORS' RIGHTS

I, the Submitting Author has the right to grant and does grant on behalf of all authors of the Work (as defined in the below author licence), an exclusive licence and/or a non-exclusive licence for contributions from authors who are: i) UK Crown employees; ii) where BMJ has agreed a CC-BY licence shall apply, and/or iii) in accordance with the terms applicable for US Federal Government officers or employees acting as part of their official duties; on a worldwide, perpetual, irrevocable, royalty-free basis to BMJ Publishing Group Ltd ("BMJ") its licensees and where the relevant Journal is co-owned by BMJ to the co-owners of the Journal, to publish the Work in BMJ Open and any other BMJ products and to exploit all rights, as set out in our licence.

## ABBREVIATIONS

BA: Behavioural Activation
CBT: Cognitive Behavioural Therapy
GRIPP2: Guidance for Reporting Patient and Public Involvement 2
LI-CBT: Low-Intensity Cognitive Behavioural Therapy
MRC: Medical Research Council
NPT: Normalization Process Theory

For peer review only

PAR: Participatory Action Research

PPI: Patient and Public Involvement

S-NoMAD: Swedish version of the Normalization Process Theory Measure

SRQR: Standards for Reporting Qualitative Research

TIDieR: Template for Intervention Description and Replication

For peer review only

## REFERENCES


1 Nichols E, Szoeke CE, Vollset SE, et al. Global, regional, and national burden of Alzheimer's disease and other dementias, 1990–2016: a systematic analysis for the Global Burden of Disease Study 2016. *Lancet Neurol* 2019;18:88-106. doi:10.1016/S1474-4422(18)30403-4.

2 Livingston G, Sommerlad A, Orgeta V, et al. Dementia prevention, intervention, and care. *Lancet* 2017;390:2673-734. doi:10.1016/S0140-6736(17)31363-6.

3 Wimo A, Jönsson L, Fratiglioni L, et al. The societal costs of dementia in Sweden 2012 – relevance and methodological challenges in valuing informal care. *Alzheimers Res Ther* 2016;8:59. doi:10.1186/s13195-016-0215-9.

4 Wright T, O'Connor S. Reviewing challenges and gaps in European and global dementia policy. *J Public Ment Health* 2018;17:157-67. doi:10.1108/JPMH-02-2018-0012.

5 Ritchie CW, Terrera GM, Quinn TJ. Dementia trials and dementia tribulations: methodological and analytical challenges in dementia research. *Alzheimers Res Ther* 2015;7:31. doi:10.1186/s13195-015-0113-6.

6 Sleeman KE, de Brito M, Etkind S, et al. The escalating global burden of serious health-related suffering: projections to 2060 by world regions, age groups, and health conditions. *Lancet Glob Health* 2019;7:e883-92. doi:10.1016/S2214-109X(19)30172-X.

7 Prince M, Acosta D, Ferri CP, et al. Dementia incidence and mortality in middle-income countries, and associations with indicators of cognitive reserve: a 10/66 dementia research group population-based cohort study. *Lancet* 2012;380:50-8. doi:10.1016/S0140-6736(12)60399-7.

8 Holopainen A, Siltanen H, Pohjanvuori A, et al. Factors associated with the quality of life of people with dementia and with quality of life-improving interventions: scoping review. *Dementia (London)* 2019;18:1507-37. doi:10.1177/1471301217716725.

9 Laver K, Dyer S, Whitehead C, et al. Interventions to delay functional decline in people with dementia: a systematic review of systematic reviews. *BMJ Open* 2016;6:e010767. doi:10.1136/bmjopen-2015- 010767.

10 Cipriani G, Lucetti C, Carlesi C, et al. Depression and dementia. A review. *Eur Geriatr Med* 2015;6:479-86. doi:10.1016/j.eurger.2015.07.010.

11 Enache D, Winblad, Aarsland D. Depression in dementia: epidemiology, mechanisms, and treatment. *Curr Opin Psychiatry* 2011;24:461-72.


For peer review only

doi:10.1097/YCO.0b013e32834bb9d4.
12 Modrego PJ. Depression in Alzheimer's Disease. Pathophysiology, diagnosis, and treatment. *J Alzheimers Dis* 2010;21:1077-87. doi:10.3233/JAD-2010-100153.
13 Steinberg M, Shao H, Zandi P, et al. Point and 5-year period prevalence of neuropsychiatric symptoms in dementia: the Cache County Study. *Int J of Geriatric Psychiatry* 2008;23:170-7. doi:10.1002/gps.1858.
14 Curran EM, Loi S. Depression and dementia. *Med J Aust* 2013;199:40-4. doi:10.5694/mjao12.10567.
15 Kitching D. Depression in dementia. *Aust prescr* 2015;38:209-11. doi:10.18773/austprescr.2015.071.
16 Orgeta V, Qazi A, Spector AE, et al. Psychological treatments for depression and anxiety in dementia and mild cognitive impairment. *Cochrane Database Syst Rev* 2014;1:CD009125. doi:10.1002/14651858.CD009125.pub2.
17 van der Roest HG, Meiland FJM, Comijs HC, et al. What do community-dwelling people with dementia need? A survey of those who are known to care and welfare services. *Int Psychogeriatr* 2009;21:949-65. doi:10.1017/S1041610209990147.
18 Kishita N, Backhouse T, Mioshi E. Nonpharmacological interventions to improve depression, anxiety, and quality of life (qol) in people with dementia: an overview of systematic reviews. *J Geriatr Psychiatry Neurol* 2019;33:28-41. doi:10.1177/0891988719856690.
19 Leyhe T, Reynolds III CF, Melcher T, et al. A common challenge in older adults: classification, overlap, and therapy of depression and dementia. *Alzheimers dement* 2017;13:59-71. doi:10.1016/j.jalz.2016.08.007.
20 Wittchen HU, Jacobi F, Rehm J, et al. The size and burden of mental disorders and other disorders of the brain in Europe 2010. *Eur Neuropsychopharmacol* 2011;21:655-79. doi:10.1016/J.EURONEURO.2011.07.018.
21 Bennett-Levy J, Richards DA, Farrand P. Low intensity CBT interventions: a revolution in mental health care. In: Bennett-Levy J, eds. Oxford guide to low intensity CBT interventions. Oxford: Oxford University Press 2010;3-18.
22 Rebello TJ, Marques A, Gureje O, et al. Innovative strategies for closing the mental health treatment gap globally. *Curr Opin Psychiatry* 2014;27:308-14. doi:10.1097/YCO.0000000000000068.
23 Farrand P. Low-intensity CBT skills and interventions: a practitioners′ manual. London: SAGE Publications Ltd 2020.

For peer review only

24 Roth A, Pilling S. The competencies required to deliver effective cognitive and behavioural therapy for people with depression and anxiety disorders. London: Department of Health 2007.

25 Farrand P, Pentecost C, Greaves C, et al. A written self-help intervention for depressed adults comparing behavioural activation combined with physical activity promotion with a self-help intervention based upon behavioural activation alone: study protocol for a parallel group pilot randomised controlled trial (BAcPAc). *Trials* 2014;15:196. doi:10.1186/1745-6215-15-196.

26 Leventhal AM. Sadness, depression, and avoidance behavior. *Behav Modif* 2008;32:759-79. doi:10.1177/0145445508317167.

27 Kerkhof Y, Pelgrum-Keurhorst M, Mangiaracina F, et al. User-participatory development of FindMyApps; a tool to help people with mild dementia find supportive apps for self-management and meaningful activities. *Digit Health* 2019;5:1-19. doi:10.1177/2055207618822942.

28 Copeland JL, Ashe MC, Biddle SJ, et al. Sedentary time in older adults: a critical review of measurement, associations with health, and interventions. *Br J Sports Med* 2017;51:1539. doi:10.1136/bjsports-2016-097210.

29 Farrand P, Woodford J, Small F, et al. Behavioural activation self-help to improve depression in people living with dementia: The PROMOTE treatment protocol. *NZ J Psychol* 2017;46:51.

30 Dodel R, Belger M, Reed C, et al. Determinants of societal costs in Alzheimer's disease: GERAS study baseline results. *Alzheimers Dement* 2015;11:933-45. doi:10.1016/J.JALZ.2015.02.005.

31 Mausbach BT, Patterson TL, Grant I. Is depression in Alzheimer's caregivers really due to activity restriction? A preliminary mediational test of the Activity Restriction Model. *J Behav Ther Exp Psychiatry* 2008;39:459-66. doi:10.1016/J.JBTEP.2007.12.001.

32 Prince M, Bryce R, Albanese E, et al. The global prevalence of dementia: a systematic review and metaanalysis. *Alzheimers Dement* 2013;9:63-75.e2. doi:10.1016/J.JALZ.2012.11.007.

33 Kasper JD, Freedman VA, Spillman BC, et al. The disproportionate impact of dementia on family and unpaid caregiving to older adults. *Health Aff* 2015;34:1642-49. doi:10.1377/hlthaff.2015.0536,

34 Bleijenberg N, de Man-van Ginkel, Janneke M, et al. Increasing value and reducing

For peer review only

waste by optimizing the development of complex interventions: Enriching the development phase of the Medical Research Council (MRC) Framework. *Int J Nurs Stud* 2018;79:86-93. doi:10.1016/j.ijnurstu.2017.12.001.

35 Craig P, Dieppe P, Macintyre S, et al. Developing and evaluating complex interventions: the new Medical Research Council guidance. *BMJ* 2008;337:a1655. doi:10.1136/bmj.a1655.

36 Heim E, Kohrt BA. Cultural adaptation of scalable psychological interventions: a new conceptual framework. *Clinical Psychology in Europe* 2019;1:1-22. doi:10.32872/cpe.v1i4.37679.

37 Castro FG, Barrera M, Holleran Steiker LK. Issues and challenges in the design of culturally adapted evidence-based interventions. *Annu Rev Clin Psychol* 2010;6:213-39. doi:10.1146/annurev-clinpsy-033109-132032.

38 Ferrer-Wreder L, Adamson L, Kumpfer KL, et al. Advancing intervention science through effectiveness research: a global perspective. *Child Youth Care Forum* 2012;41:109-17. doi:10.1007/s10566-012-9173-y.

39 Brantnell A, Woodford J, Baraldi E, et al. Views of implementers and nonimplementers of internet-administered cognitive behavioral therapy for depression and anxiety: survey of primary care decision makers in Sweden. *J Med Internet Res* 2020;22:e18033. doi:10.2196/18033.

40 Bach-Mortensen AM, Lange BCL, Montgomery P. Barriers and facilitators to implementing evidence-based interventions among third sector organisations: a systematic review. *Implement Sci* 2018;13:103-19. doi:10.1186/s13012-018-0789-7.

41 Murray E, Treweek S, Pope C, et al. Normalisation process theory: a framework for developing, evaluating and implementing complex interventions. *BMC Med* 2010;8:63. doi:10.1186/1741-7015-8-63.

42 May C, Finch T. Implementing, embedding, and integrating practices: an outline of normalization process theory. *Sociology* 2009;43:535-54. doi:10.1177/0038038509103208.

43 May CR, Cummings A, Girling M, et al. Using normalization process theory in feasibility studies and process evaluations of complex healthcare interventions: a systematic review. *Implement Sci* 2018;13:80. doi:10.1186/s13012-018-0758-1.

44 May C, Rapley T, Mair FS, et al. Normalization process theory on-line users' manual, toolkit and NoMAD instrument. 2015. Available from: http://www.normalizationprocess.org. [Cited 2020 Oct 14].

For peer review only

45 McEvoy R, Ballini L, Maltoni S, et al. A qualitative systematic review of studies using the normalization process theory to research implementation processes. *Implement Sci* 2014;9:2. doi:10.1186/1748-5908-9-2.
46 Mills K, Griffin SJ, Sutton S, et al. Development and usability testing of a very brief intervention for personalised cancer risk assessment to promote behaviour change in primary care using normalisation process theory. *Prim Health Care Res Dev* 2020;21:e1. doi:10.1017/S146342361900080X.
47 Early F, Wilson P, Deaton C, et al. Developing an intervention to increase REferral and uptake TO pulmonary REhabilitation in primary care in patients with chronic obstructive pulmonary disease (the REsTORE study): mixed methods study protocol. *BMJ Open* 2019;9:e024806. doi:10.1136/bmjopen-2018-024806.
48 Goeman D, Michael J, King J, et al. Partnering with consumers to develop and evaluate a Vietnamese Dementia Talking-Book to support low health literacy: a qualitative study incorporating. *BMJ Open* 2016;6:e011451. doi:10.1136/bmjopen-2016-011451.
49 Sandlund M, Lindgren H, Pohl P, et al. Towards a mobile exercise application to prevent falls: a participatory design process. *Int J Child Health Hum Dev* 2016;9:389-98.
50 Nygren M, Roback K, Öhrn A, et al. Factors influencing patient safety in Sweden: perceptions of patient safety officers in the county councils. *BMC Health Serv Res* 2013;13:52. doi:10.1186/1472-6963-13-52.
51 Socialtjänstlag (SFS 2001:453). Stockholm: Socialdepartementet. Available from: https://www.riksdagen.se/sv/dokument-lagar/dokument/svensk-forfattningssamling/socialtjanstlag-2001453_sfs-2001-453. [Cited 2020 July 14].
52 Statistiska centralbyrån. Folkmängd i riket, län och kommuner 30 september 2019 och befolkningsförändringar 1 januari–30 september 2019. Stockholm: Statistiska centralbyrån; 2019. Available from: https://www.scb.se/hitta-statistik/statistik-efter-amne/befolkning/befolkningens-sammansattning/befolkningsstatistik/pong/tabell-och-diagram/kvartals--och-halvarsstatistik--kommun-lan-och-riket/kvartal-13-2019/. [Cited 2020 Feb 6].
53 Region Uppsala. Demenssjukdomar- kliniska riktlinjer. Uppsala: Region Uppsala; 2018. Available from: http://publikdocplus.regionuppsala.se/Home/GetDocument?containerName=e0c73411-be4b-4fee-ac09-640f9e2c5d83&reference=DocPlusSTYR-

4202&docId=DocPlusSTYR-4202&filename=Demenssjukdomar%20-%20kliniska%20riktlinjer.pdf. [Cited 2020 Feb 6].

54 O'Connor S, Hanlon P, O'Donnell CA, et al. Understanding factors affecting patient and public engagement and recruitment to digital health interventions: a systematic review of qualitative studies. *BMC Med Inform Decis Mak* 2016;1:120. doi:10.1186/s12911-016-0359-3.

55 Hälso- och sjukvårdslag (SFS 2017:30). Stockholm: Socialdepartementet. Available from: https://www.riksdagen.se/sv/dokument-lagar/dokument/svensk-forfattningssamling/halso--och-sjukvardslag_sfs-2017-30. [Cited 2020 July 14].

56 Warner J, McCarney R, Griffin M, et al. Participation in dementia research: rates and correlates of capacity to give informed consent. *J Med Ethics* 2008;34:167-70. doi:10.1136/jme.2006.019786.

57 Dementia Engagement and Empowerment Project. Writing dementia-friendly information. Exeter: Dementia Engagement and Empowerment Project; 2013. Available from: https://www.dementiavoices.org.uk/wp-content/uploads/2013/11/DEEP-Guide-Writing-dementia-friendly-information.pdf. [Cited 2019 Oct 21].

58 Barnes M, Wiles N, Morrison J, et al. Exploring patients' reasons for declining contact in a cognitive behavioural therapy randomised controlled trial in primary care. *Br J Gen Pract* 2012;62:e371-7. doi:10.3399/bjgp12X641492.

59 Farrand P, Woodford J, Llewellyn D, et al. Behavioural activation written self-help to improve mood, wellbeing and quality of life in people with dementia supported by informal carers (PROMOTE): a study protocol for a single-arm feasibility study. *Pilot Feasibility Stud* 2016;2:42. doi:10.1186/s40814-016-0083-x.

60 Woodford J, Farrand P, Watkins ER, et al. Supported cognitive-behavioural self-help versus treatment-as-usual for depressed informal carers of stroke survivors (CEDArS): study protocol for a feasibility randomized controlled trial. *Trials* 2014;15:157. doi:10.1186/1745-6215-15-157.

61 Heslop CW, Burns S, Lobo R, et al. Developing a framework for community-based sexual health interventions for youth in the rural setting: protocol for a participatory action research study. *BMJ Open* 2017;7:e013368. doi:10.1136/bmjopen-2016-013368.

62 Elf M, Nordmark S, Lyhagen J, et al. The Swedish version of the Normalization Process Theory Measure S-NoMAD: translation, adaptation, and pilot testing.

For peer review only

*Implement Sci* 2018;13:146. doi:10.1186/s13012-018-0835-5.

63 Elo S, Kyngäs H. The qualitative content analysis process. *J Adv Nurs* 2008;62:107-15. doi:10.1111/j.1365-2648.2007.04569.x.

64 Elo S, Kääriäinen M, Kanste O, et al. Qualitative content analysis: a focus on trustworthiness. *SAGE Open* 2014;4:1-10. doi:10.1177/2158244014522633.

65 Krefting L. Rigor in qualitative research: the assessment of trustworthiness. *Am J Occup Ther* 1991;45:214–22. doi:10.5014/ajot.45.3.214.

66 O'Brien BC, Harris IB, Beckman TJ, et al. Standards for reporting qualitative research: a synthesis of recommendations. *Acad Med* 2014;89:1245-51. doi:10.1097/ACM.0000000000000388.

67 Hoffmann TC, Glasziou PP, Boutron I, et al. Better reporting of interventions: template for intervention description and replication (TIDieR) checklist and guide. *BMJ* 2014;348:g1687. doi:10.1136/bmj.g1687.

68 Staniszewska S, Brett J, Simera I, et al. GRIPP2 reporting checklists: tools to improve reporting of patient and public involvement in research. *BMJ* 2017;358:j3453. doi:10.1136/bmj.j3453.

69 Mann C, Chilcott S, Plumb K, et al. Reporting and appraising the context, process and impact of PPI on contributors, researchers and the trial during a randomised controlled trial - the 3D study. *Res Involv Engagem* 2018;4:15. doi:10.1186/s40900-018-0098-y.

70 Socialstyrelsen. Nationella riktlinjer för vård och omsorg vid demenssjukdom: stöd för styrning och ledning. Stockholm: Socialstyrelsen; 2017. Available from: https://www.socialstyrelsen.se/globalassets/sharepoint-dokument/artikelkatalog/nationella-riktlinjer/2017-12-2.pdf. [Cited 2019 Jul 15].

71 World Medical Association. World medical association declaration of helsinki: Ethical principles for medical research involving human subjects. *JAMA* 2013;310:2191-4. doi:10.1001/jama.2013.281053.

72 European Parliament and the Council of the European Union. Regulation (EU) 2016/679 of the European Parliament and of the Council of 27 April 2016 on the protection of natural persons with regard to the processing of personal data and on the free movement of such data, and repealing Directive 95/46. *OJEU* 2016;59:1-88.

73 Lag om etikprövning av forskning som avser människor (SFS 2004:460). Stockholm: Utbildningsdepartementet. Available from: https://www.riksdagen.se/sv/dokument-lagar/dokument/svensk-forfattningssamling/lag-2003460-om-etikprovning-av-forskning-som_sfs-2003-460. [Cited 2020 July 14].

BMJ Open

# Adapting a guided low-intensity behavioural activation intervention for people with dementia and depression in the Swedish healthcare context (INVOLVERA): a study protocol utilising co-design and participatory action research

| | |
|---|---|
| Journal: | *BMJ Open* |
| Manuscript ID | bmjopen-2020-048097.R1 |
| Article Type: | Protocol |
| Date Submitted by the Author: | 02-Jun-2021 |
| Complete List of Authors: | Svedin, Frida; Uppsala University, Clinical Psychology in Healthcare, Department of Women's and Children's Health<br>Brantnell, Anders; Uppsala University, Clinical Psychology in Healthcare, Department of Children's and Women's Health; Uppsala University, Division of Industrial Engineering and Management, Department of Civil and Industrial Engineering<br>Farrand, Paul; University of Exeter, Clinical Education Development and Research (CEDAR)<br>Blomberg, Oscar; Uppsala University, Clinical Psychology in Healthcare, Department of Women's and Children's Health<br>Coumoundouros, Chelsea; Uppsala University, Clinical Psychology in Healthcare, Department of Women's and Children's Health<br>von Essen, Louise; Uppsala University, Clinical Psychology in Healthcare, Department of Women’s and Children’s Health<br>Åberg, Anna ; Uppsala University, Geriatrics, Department of Public Health and Caring Sciences; Dalarna University, Medical Science, School of Education, Health and Social Studies<br>Woodford, Joanne; Uppsala University, Clinical Psychology in Healthcare, Department of Women's and Children's Health |
| <b>Primary Subject Heading</b>: | Mental health |
| Secondary Subject Heading: | Health services research, Public health, Qualitative research, Geriatric medicine |
| Keywords: | Dementia < NEUROLOGY, MENTAL HEALTH, GERIATRIC MEDICINE, Depression & mood disorders < PSYCHIATRY, PUBLIC HEALTH, PSYCHIATRY |
| | |

SCHOLARONE™
Manuscripts

*I, the Submitting Author has the right to grant and does grant on behalf of all authors of the Work (as defined in the below author licence), an exclusive licence and/or a non-exclusive licence for contributions from authors who are: i) UK Crown employees; ii) where BMJ has agreed a CC-BY licence shall apply, and/or iii) in accordance with the terms applicable for US Federal Government officers or employees acting as part of their official duties; on a worldwide, perpetual, irrevocable, royalty-free basis to BMJ Publishing Group Ltd ("BMJ") its licensees and where the relevant Journal is co-owned by BMJ to the co-owners of the Journal, to publish the Work in this journal and any other BMJ products and to exploit all rights, as set out in our licence.*

*The Submitting Author accepts and understands that any supply made under these terms is made by BMJ to the Submitting Author unless you are acting as an employee on behalf of your employer or a postgraduate student of an affiliated institution which is paying any applicable article publishing charge ("APC") for Open Access articles. Where the Submitting Author wishes to make the Work available on an Open Access basis (and intends to pay the relevant APC), the terms of reuse of such Open Access shall be governed by a Creative Commons licence – details of these licences and which Creative Commons licence will apply to this Work are set out in our licence referred to above.*

*Other than as permitted in any relevant BMJ Author's Self Archiving Policies, I confirm this Work has not been accepted for publication elsewhere, is not being considered for publication elsewhere and does not duplicate material already published. I confirm all authors consent to publication of this Work and authorise the granting of this licence.*

For peer review only

# Adapting a guided low-intensity behavioural activation intervention for people with dementia and depression in the Swedish healthcare context (INVOLVERA): a study protocol utilising co-design and participatory action research

Frida SVEDIN[1] (frida.svedin@kbh.uu.se), Anders BRANTNELL[1,2] (anders.brantnell@kbh.uu.se), Paul FARRAND[3] (p.a.farrand@exeter.ac.uk), Oscar BLOMBERG[1] (oscar.blomberg@kbh.uu.se), Chelsea COUMOUNDOUROS[1] (chelsea.coumoundouros@kbh.uu.se), Louise von ESSEN[1] (louise-von.essen@kbh.uu.se), Anna Cristina ÅBERG[4,5] (anna.cristina.aberg@pubcare.uu.se), Joanne WOODFORD[1] (joanne.woodford@kbh.uu.se)

**Corresponding author**

Joanne Woodford, Clinical Psychology in Healthcare, Department of Women's and Children's Health, Uppsala University, 751 85 Uppsala, Sweden. E-mail: joanne.woodford@kbh.uu.se. Telephone: 0046 729 999 211

**Affiliations**

[1]Clinical Psychology in Healthcare, Department of Women's and Children's Health, Uppsala University, 751 85 Uppsala, Sweden.

[2]Division of Industrial Engineering and Management, Department of Civil and Industrial Engineering, Uppsala University, 751 21 Uppsala, Sweden.

[3]Clinical Psychology, Education, Development and Research (CEDAR), Psychology, University of Exeter, Perry Road, Devon, EX4 4QG, United Kingdom.

[4]Geriatrics, Department of Public Health and Caring Sciences, Uppsala University, 751 85 Uppsala, Sweden.

For peer review only

[5]Medical Science, School of Education, Health and Social Studies, Dalarna University, 791 88 Falun, Sweden.

**Word count: 4744 (excluding: title page, abstract, acknowledgements, contributions, and references)**

For peer review only

## ABSTRACT

**Introduction:** Dementia is a worldwide health concern with incident rates continuing to increase. Whilst depression prevalence is high in people with dementia, and psychological interventions such as cognitive behavioral therapy are effective, access to psychological interventions remains limited. Reliance on traditional cognitive behavioral therapy for people with dementia and depression may present difficulties given it is a complex psychological approach, costly to deliver, and professional training time is lengthy. An alternative approach is behavioural activation, a simpler psychological intervention for depression. The present study seeks to work with people with dementia, informal caregivers, community stakeholders, and healthcare professionals, to adapt a guided low-intensity behavioural activation intervention for people with dementia and depression whilst maximising implementation potential within the Swedish healthcare context.

**Methods and analysis:** A mixed-methods study utilising Co-Design, principles from Participatory Action Research (PAR), and Normalisation Process Theory (NPT) to facilitate the cultural relevance, appropriateness, and implementation potential of the intervention. The study will consist of four iterative PAR phases, using focus groups with healthcare professionals and community stakeholders, and semi-structured interviews with people with dementia and informal caregivers. A content analysis approach will be adopted to analyse the transcribed focus groups and semi-structured interviews recordings.

**Ethics and dissemination:** The study will be conducted in accordance with the Helsinki Declaration and data handled according to General Data Protection Regulation. Written informed consent will be obtained from all study participants. In accordance with the Swedish Health and Medical Services Act, capacity to consent will be examined by a member of the research team. Ethical approval has been obtained from the Swedish Ethical Review Authority (Dnr: 2020-05542 and Dnr: 2021-00925). Findings will be published in an open access peer-reviewed journal, presented at academic conferences, and disseminated amongst lay and healthcare professional audiences.

For peer review only

## STRENGTHS AND LIMITATIONS OF THIS STUDY

- To the best of our knowledge, this is the first study in Sweden integrating Co-Design, principles from Participatory Action Research, and Normalisation Process Theory to develop a psychological intervention for depression in people with dementia.
- Including community stakeholders and healthcare professionals can help ensure the intervention is truly clinic- and patient-centered due to their 'real-world' knowledge and ability to provide useful information concerning how to efficiently implement the intervention.
- Utilisation of implementation theory i.e. Normalisation Process Theory should improve implementation potential and potentially optimise effectiveness.
- People with dementia do not need to have past or present depression to participate, hence we may recruit people with dementia who have no experience of depression or low mood, which may impact on transferability or results.
- With limited resourses, this study will not be able to include ethnic minority groups due to the increasing costs for e.g., recruitment, informed consent, and need for translated materials.

**KEY WORDS**

Dementia, Depression, Co-Design, Participatory Action Research, Normalisation Process Theory, Cultural adaptation

For peer review only

# INTRODUCTION

## Background

Dementia is a worldwide health concern, with the number of people living with dementia set to rise from approximately 44 million in 2016[1] to over 140 million by 2050.[2] In Sweden, over 140,000 people are living with dementia,[1] with around 25,000 incident cases each year.[3] Further, the societal cost of dementia in Sweden is high and was estimated at nearly 63 billion SEK during 2012.[3] Due to both prevalence rates and the significant burden placed on individuals and wider society, dementia represents a challenge for global healthcare policy,[4] especially given the lack of curative or preventative medical interventions.[5] Indeed, dementia is expected to represent the largest increase in serious health-related suffering and burden globally between 2016-2060,[6] and is associated with increased mortality,[7] poor quality of life,[8] and poor functional outcomes.[9] Further contributing to health-related suffering are elevated symptoms of depression, with prevalence rates estimated to be between 30-87%.[10-13] Depression in people with dementia is associated with increased mortality,[14] poor quality of life,[10] sleep difficulties,[15] and reduction in activities of daily living.[10] Despite depression being highly prevalent, and evidence-based psychological interventions such as cognitive behavioral therapy (CBT) being effective for people with mild-to-moderate dementia,[16] access to evidence-based psychological interventions remains limited.[17] Lack of access is of particular importance since pharmacological approaches for depression show poor efficacy and negative side effects in people with dementia.[18-19]

However, this psychological 'treatment gap' is not unique to the dementia population, with only 30% of people across Europe with mental health difficulties accessing support.[20] To address the psychological treatment gap, there have been global efforts to increase access to psychological interventions through the provision of Low-Intensity CBT (LI-CBT).[21-22] LI-CBT can be characterised as a single-strand approach, adopting a single CBT technique to target specific common mental health difficulties where an evidence base has been demonstrated.[23] Further, LI-CBT is delivered in a self-help format via a health technology (e.g., written workbooks, audio books, internet-administered, smartphone applications),[21] with the provision of face-to-face, telephone, or e-mail guidance associated with larger effect sizes.[23] For example, across England, guidance to LI-CBT is provided by a psychological practitioner workforce [23] with graduate or postgraduate level training in the competencies required to support LI-CBT.[24]

For peer review only

LI-CBT may represent a solution to improve access to support for people with dementia and depression. One evidence based LI-CBT technique for depression is simple behavioural activation (BA). The simple BA model seeks to target behavioural avoidance (e.g., disengagement from pleasurable, routine, and necessary activities),[25] a common symptom of both depression[26] and dementia,[27] by increasing engagement in activities. As such, BA may also help overcome sedentary behaviours common in older adults,[28] which may lead to improved functioning in people with dementia (e.g., engagement in, and ability to perform, activities of daily life). Further, the simple BA model is a straightforward psychological approach[29] and may be easier for people with dementia to understand than more complex CBT techniques. In addition to showing promise for people with dementia and depression, BA may also benefit informal caregivers[30] given improved functioning in the person with dementia is associated with decreases in caregiver burden.[30] Informal caregivers may also engage more in activities with the person with dementia, possibly improving caregivers' own mental health.[31] This is of particular importance given global and social care priorities to support people with dementia to remain at home for as long as possible[32] have resulted in an increased reliance on support from informal caregivers.[33]

Given the promise of BA, a research programme, informed by Phase I (development) of the Medical Research Council (MRC) complex interventions framework,[34-35] has been undertaken in England.[29] However, before applying the intervention in Sweden, there is a need to adapt the intervention to fit into the Swedish cultural[36] and healthcare context[37] whilst maintaining fidelity to the intervention.[38] Cultural adaptation of an intervention refers to the systematic modification of the intervention considering language, culture, and context to ensure its compatibility with the target population's cultural patterns, meanings, and values.[39] An example of a language differences is the meaning of the term 'depressed', which in Swedish is associated with a formal diagnosis of depression, whereas in English the term is used more colloquially.[40] Consideration of context can include a number of characteristics that could impact intervention delivery and effectiveness, and may include geographical, sociocultural, socioeconomic, ethical, legal, and political factors.[41-42] One contextual difference between England and Sweden is at present there is no psychological practitioner workforce in Sweden specifically trained in supporting LI-CBT.[43] As such, an appropriate healthcare professional workforce will need to be identified to facilitate intervention delivery. In addition, in England national non-profit organisations, such as

For peer review only - http://bmjopen.bmj.com/site/about/guidelines.xhtml

Alzheimer's societies, provide support such as cognitive rehabilitation, information for informal caregivers, and activity groups, whereas in Sweden, support is predominantly provided via formal health and social care services rather than non-governmental organisations.[44] However, to the best of our knowledge, little research has examined potential cultural, language, and contextual differences between England and Sweden in the context of psychological intervention development and adaptation. Consequently, it is difficult to anticipate in advance what cultural adaptations may be required.

In addition, it is increasingly recognised that there are multiple facilitators and barriers to the implementation of evidence-based interventions, e.g., problems with adaptation, fidelity, and delivery capability.[45] Normalisation Process Theory (NPT) provides a framework for understanding key mechanisms that facilitate or hinder the implementation of a complex intervention.[46-48] NPT proposes four key constructs that enable the normalisation of a complex intervention by stakeholders: (1) Coherence (individual and collective sense-making); (2) Cognitive participation (engagement to build and sustain practice); (3) Collective action (enact practices); and (4) Reflective monitoring (appraisal).[47, 49] Traditionally, NPT has been used to analyse the implementation of complex interventions.[50] However, more recently NPT has been used to inform the development of complex interventions to enhance implementation potential.[51-52] In the present study, we will use NPT throughout the adaptation process to gain an understanding of what may facilitate or hinder the implementation of the intervention and identify implementable evidence-based methods of intervention delivery.

**Aim and objectives**

The overall aim is to adapt the guided Low-Intensity BA intervention developed in England for people with dementia and depression and their informal caregivers, and enhance implementation potential for the Swedish cultural and healthcare context. Objectives are to:

1. Develop an understanding of the existing healthcare and community support context for people with dementia and their informal caregivers;
2. Identify barriers and facilitators to intervention uptake;
3. Identify feasible, acceptable, and implementable evidence-based methods of intervention delivery informed by NPT;
4. Adapt the guided Low-Intensity BA intervention to ensure cultural appropriateness, relevancy, and acceptability to people with dementia and their informal caregivers.

For peer review only

## METHODS AND ANALYSIS

### Study design

A mixed-methods study utilising Co-Design and principles from Participatory Action Research (PAR)[53-54], placing key stakeholders (e.g., people with dementia, informal caregivers, healthcare professionals, and community stakeholders) at the centre of the research process.[53] The study commenced 23 March 2021 and the planned end date is 23 March 2022.

### Setting

Sweden is divided into 290 municipalities and 21 regions. Regions are predominately responsible for general healthcare,[55] with municipalities responsible for general and specialised care and services in the home, nursing homes, day care, respite care, and support for informal caregivers.[56] This study will seek to involve key stakeholders from both general and specialised health and social care services. People with dementia and informal caregivers will be recruited via four specialised health and social care service settings across the county of Uppsala: Primary Healthcare Centres; Memory Clinic; Day Care Centres; and via Dementia Care Consultants. Uppsala county incudes both rural and urban areas, and approximately 390,000 residents[57] with more than 5,000 people estimated having a diagnosis of dementia.[58] Healthcare professionals and community stakeholders from non-profit organisations will be recruited from locations across Sweden.

### Study participants

Healthcare professionals and community stakeholders

Healthcare professionals (n≈8) e.g., primary care and hospital based physicians involved in dementia diagnosis, dementia care consultants, memory clinic nurses, nursing assistants working with people with dementia in day care centre settings, and clinical psychologists will be recruited. Community stakeholders (n≈8) from relevant non-profit organisations will also be recruited, e.g., from Dementia League, Alzheimer Sweden, and Red Cross.

People with dementia and informal caregivers

The following inclusion criteria will be applied for people with dementia (n≈10):

For peer review only

1. Have a self-reported diagnosis of dementia (any type). The research team will not confirm the diagnosis by checking medical records;
2. Live at home;
3. Able to and have the capacity to provide informed consent, indicating mild to moderate dementia;[59]
4. Able to speak and understand Swedish.

People with dementia will be excluded if they have:

1. A self-reported diagnosis of a severe and enduring mental health difficulty (e.g., psychosis, type I or II bipolar disorder, and personality disorder);
2. A visual or auditory impairment that would hinder their ability to participate in semi-structured interviews and/or give feedback on the intervention;
3. A self-reported misuse of alcohol or prescription or street drugs reported by the potential participant as so severe that it interferes with their ability to perform normal activities in daily life.

The following inclusion criteria will be applied for informal caregivers (n≈10):

1. Self-identified informal caregiver of a person with dementia with whom they have regular contact (at least weekly);
2. 18 years of age or older;
3. Able to speak, understand, and write in Swedish.

Informal caregivers will be excluded if they have:

1. A self-reported diagnosis of a severe and enduring mental health difficulty (e.g. psychosis, type I or II bipolar disorder, and personality disorder);
2. A visual or auditory impairment that would hinder their ability to participate in semi-structured interviews and/or give feedback on the intervention;
3. A self-reported misuse of alcohol or prescription or street drugs reported by the potential participant as so severe that it interferes with their ability to perform normal activities in daily life.

**Sample size considerations**

Sample size will be guided by thematic data saturation and as such we cannot stipulate the exact sample size in advance.[60-61] Interviews and focus groups will be analysed iteratively and a decision concerning whether thematic saturation is met will be made during analysis.

For peer review only

However, we anticipate to include n≈8 healthcare professionals, n≈8 community stakeholders in each focus group[62], and n≈10 people with dementia, and n≈10 informal caregivers respectively to participate in semi-structured interviews.

**Recruitment**

Healthcare professionals and community stakeholders

Healthcare professionals and community stakeholders will be identified via in person-networks, social media, posters on notice boards, and printed flyers.[63] A member of the research team will speak to interested stakeholders over the telephone to provide more information alongside hand out a study invitation pack by post or e-mail containing a: (1) study invitation letter; (2) study information sheet; (3) reply slip; (4) reasons for non-participation questionnaire; and (5) stamped addressed envelope.

People with dementia and informal caregivers

A multifaceted approach to recruitment will be adopted across four settings:

*Primary healthcare centres*

Primary healthcare centres who are part of the network of Academic Primary Healthcare Centres will appoint a research nurse to conduct patient record searches to identify potentially suitable people with dementia. When identified, a research nurse will make contact and ask verbal permission, face-to-face or via telephone, for a member of the research team to contact them. If permission is granted, a member of the research team will contact the person with dementia over the telephone to provide more study information and send a study invitation pack (as above) by post to interested potential participants. The study invitation pack will also include an envelope containing a separate study invitation pack to be passed on to an informal caregiver.

*Memory clinic*

A physician will provide people with dementia and/or informal caregivers with brief verbal study information alongside a study invitation pack (as above) during meetings whereby a person receives a diagnosis of dementia or follow-up visits (≤12 months since diagnosis).

*Day care centres*

For peer review only

Day care centre staff will provide informal caregivers with brief verbal study information and a study invitation pack (as above).

*Dementia care consultants*

Dementia care consultants will provide people with dementia and/or informal caregivers with brief verbal study information alongside a study invitation pack (as above).

**Informed consent and eligibility screening**

Healthcare professionals and community stakeholders

Interested healthcare professionals and community stakeholders respond to the study invitation by returning a reply slip, telephoning, or e-mailing the research team. A member of the research team will speak to those expressing interest in participation. During this phone call, interested stakeholders will have the opportunity to ask further questions about the study. Written informed consent will be obtained either face-to-face or via the post, or alternatively, verbal recorded consent will be obtained over the telephone, or via secure videoconferencing system. After provision of informed consent, background and socio-demographic characteristics—age, gender, profession, length of time in profession, professional qualifications, and length of time working with dementia – will be collected.

People with dementia and informal caregivers

Interested people with dementia and informal caregivers can respond to the research team by returning a reply slip, telephoning, or e-mailing the research team. Those expressing interest in participation will be contacted by a member of the research team who will arrange a meeting (either face-to-face, over the telephone, or via secure videoconferencing system) to gain informed consent and undertake an eligibility screen. In accordance with the Swedish Health and Medical Services Act,[64] capacity to consent in people with dementia will be assessed by a member of the research team, with appropriate knowledge and training, at the meeting. People with dementia will be provided with appropriate support to maximise ability to provide informed consent,[65] for example the provision of dementia-friendly written information.[66] Written informed consent will be obtained for people with dementia and informal caregivers met face-to-face and verbal recorded informed consent will be obtained by those met over telephone or secure videoconferencing.

After provision of informed consent, an eligibility screening will take place with questions

For peer review only

asked in accordance with the inclusion/exclusion criteria. Given people with dementia may find it difficult to provide this data, an informal caregiver will be asked to attend the meeting and provide proxy data if needed.

**Reasons for non-participation**

Study invitation packs will include an anonymous reasons for non-participation form for those who decline participation. The reasons for non-participation form consists of a short questionnaire including possible reasons for non-participation informed by previous research[67-68] and an open-ended question. Reasons for non-participation will provide important information regarding recruitment feasibility and the acceptability of the proposed intervention.[68-69]

**Intervention**

The full clinical protocol for the Low-Intensity BA intervention developed for people with dementia in England can be found elsewhere.[29] Therapeutic content follows a simple BA intervention protocol.[21] Activities are categorised into three types: (1) routine (activities providing life structure, e.g., housework); (2) pleasurable (activities providing a sense of pleasure or enjoyment determined by the person with dementia); and (3) necessary (activities that if not done, have the potential for a serious negative consequences e.g., taking medication, or paying a bill). People with dementia are supported to either re-engage with activities they have stopped doing, or replace activities they are no longer able to do (e.g., due to symptoms of dementia) with activities of similar value, importance, and meaning. Re-engaging in activity is done in a structured and gradual manner, with an overall aim to establish a balance of activities. The simple BA protocol includes four main steps (identifying current activities, identifying stopped activities or new activities, organising activities, and planning activities). Two workbooks are provided to help support intervention delivery. One workbook[70] is designed for the person with dementia to work through the steps of BA. The second workbook[71] is designed for an informal caregiver to help support the person with dementia work through the steps of BA. The overall model of delivery is shown in Figure 1. The intervention is delivered using a maximum of 12 sessions over a period of 3 months. The intervention is guided by healthcare professionals who have been trained in the competencies needed to support CBT self-help[21, 72] and an informal caregiver also provides support to the person with dementia to work through the intervention. Session One (Assessment) and

Session Two (Setting Up Support) are attended by the healthcare professional, person with dementia, and informal caregiver. Thereafter, the informal caregiver receives weekly telephone support 'check-ins' with the healthcare professional to review difficulties, intervention progress, and problem solve, and plan the next steps. The final session (Relapse Prevention) is attended by the healthcare professional, person with dementia, and informal caregiver.

- INSERT FIGURE 1 ABOUT HERE –

**PAR Phases**

Four PAR Phases will be conducted, with estimated time frame of one month for each Phase. An overview of the data collection procedure and topics for the four PAR Phases can be found in Figure 2.

- INSERT FIGURE 2 ABOUT HERE -

PAR Phase One

Two focus group meetings (90-120 minutes) will be held with healthcare professionals and community stakeholders, with semi-structured interviews offered to those unable to attend a focus group. Two members from the research team will organise and facilitate the focus groups, one as a moderator and one as an observer. Prior to the focus group/interview, participants will be provided with a written summary of the model of delivery developed in England and translated copies of the two workbooks in Swedish.

A topic guide, informed by NPT[47, 49] will consist of open-ended questions examining:

1. Perceived purpose of the intervention;
2. First impressions of the intervention;
3. Potential impact of the intervention on people with dementia and informal caregivers;
4. Perceived differences and similarities to existing support available for people with dementia and informal caregivers;

For peer review only

5. Barriers and facilitators to people with dementia and informal caregivers using the intervention (e.g., travel and mobility, physical health problems, stigma, informal caregiver resistance, COVID-19 restrictions);
6. Potential professional/non-professional group to support and guide people with dementia and informal caregivers using the intervention (e.g., dementia care consultants, nurses, psychologists, volunteers);
7. Preferred intervention delivery setting (e.g., home, primary healthcare centre, memory clinic, day care);
8. Type of support and guidance (e.g., telephone, face-to-face, online) needed to facilitate people with dementia and informal caregivers using the intervention; and
9. Anticipated barriers and facilitators to the potential professional/non-professional group implementing the intervention (e.g., resources, training, managerial support, change agents).

Focus groups/interviews may be held face-to-face, via videoconference, or over the telephone, dependent on preference, and will be audio recorded. The research team will keep a reflective journal, logging the progresses, obstacles, and successes of the research process.[73]

PAR Phase Two

Individual semi-structured interviews will be held with people with dementia and informal caregivers respectively. Prior to the interview, participants will be provided with a written summary of the model of delivery developed in England and translated copies of the two workbooks in Swedish. A topic guide, informed by NPT[47, 49] will examine similar topics to those examined in PAR Phase One. Additional focus will be placed on the perceived relevance of the intervention content and language and ways to enhance relevancy, cultural appropriateness, and acceptability. Interviews (45-60 minutes) will be held face-to-face (in a convenient location for the person with dementia and informal caregiver, e.g., at their home or other familiar community location chosen by the participant), or via videoconference/telephone.

PAR Phase Three

Based on PAR Phase One and Two results, a new version of the intervention and proposed model of delivery (e.g., professional/non-professional group to support and guide people with dementia and informal caregivers using the intervention, intervention delivery and type of

For peer review only

support and guidance) will be developed. Two focus group meetings will be held with healthcare professionals and community stakeholders from PAR Phase One, with semi-structured interviews offered to those unable to attend. Two members from the research team will organise and facilitate the focus groups, one as a moderator and one as an observer. A topic guide, similar to the one used in PAR Phase One will guide focus groups/interviews and examine the implementation potential of the new version of the intervention in accordance with NPT. At the end of the focus groups/interviews, the Swedish version of the NPT Measure (S-NoMAD) [74] will be completed by all healthcare professionals to examine implementation potential. The S-NoMAD[74] consists of 20 items corresponding to the four NPT constructs (Coherence, Cognitive Participation, Collective Action, and Reflexive Monitoring)[47] in relation to the potential implementation of the proposed BA intervention. Responses are provided using a five-point Likert scale, ranging from "Strongly Disagree" to "Strongly Agree". "Neutral" and "Not applicable" are also given as options.[74] The internal consistency of the S-NoMAD (Cronbach's alpha 0.76 to 0.83) are in line with the original NoMAD.[75]

PAR Phase Four

A second round of semi-structured interviews with the same people with dementia and informal caregivers who participated in PAR Phase Two will be conducted respectively. A new version of the intervention and proposed delivery model established in PAR Phases One-Three will be presented. A topic guide, similar to the one used in PAR Phase Two, will guide interviews and examine the implementation potential of the new version of the intervention in accordance with NPT.

**Data analysis**

Focus groups/interviews will be transcribed and uploaded into NVivo version 12.0 to support data analysis. Data analysis will be iterative, e.g., will be conducted after each PAR Phase to inform the subsequent phase. A content analysis approach will be adopted to analyse transcribed recordings.[76] NPT constructs will inform deductive coding, however inductive coding will be applied to relevant data unrelated to NPT constructs. The S-NoMAD results will be analysed descriptively[74] in SPSS version 25. Triangulation by data source, where data sources are assessed against one another to cross-check data and aid interpretation,[77] will be conducted by exploring agreements and disagreements across the qualitative (interviews/focus groups) and quantitative (S-NoMAD) data. Comparisons between the

For peer review only

qualitative and quantitative data sets will be discussed in the research team to explore potential reasons for discordant data.

## Trustworthiness

Trustworthiness will be established via the use of a trustworthiness checklist developed for content analysis.[78] To ensure study credibility, peer examination (e.g., researchers discussing the research process and findings with impartial and experienced colleagues), field journals, and member checking (e.g., continuously testing with informants themes, interpretations, and conclusions) will be conducted. Dense description and presenting demographic data will be incorporated to ensure transferability.[77] Two members of the research team will independently identify codes and themes,[77] and thereafter compare their findings. Codes and themes will also be discussed by the wider research team and Patient and Public Involvement group (see section on Patient and Public Involvement).

## Reporting

Qualitative results will be reported through the Standards for Reporting Qualitative Research (SRQR).[79] Intervention content will be reported in accordance with the Template for Intervention Description and Replication (TIDieR).[80] Patient and Public involvement will follow Guidance for Reporting Patient and Public Involvement (GRIPP2).[81]

## Patient and public involvement

A Patient and Public Involvement (PPI) group consisting of people with dementia (n=3) and informal caregivers (n=3) will be established to work alongside the research team as active research partners throughout the research process. The PPI group will be recruited via advertisement through existing networks, relevant online/social media groups, and relevant local healthcare settings. The PPI group will meet together with the research team once every two-three months, with meetings held face-to-face, via video conference and/or telephone depending on group members' preferences. The PPI group will work alongside the research team with research activities, such as advising on recruitment and research procedures, co-development of the intervention materials, interpretation of study results, and dissemination. A member of the research team will log all PPI activities in an impact log, which will be distributed to PPI group members immediately after each meeting to comment on accuracy.[82]

For peer review only

## DISCUSSION

At present, the psychological support needs of people with dementia and depression living at home are not met by the Swedish healthcare system.[83] To the best of our knowledge, this will be the first study to develop a Low-Intensity BA intervention for people with dementia in the Swedish setting. The proposed intervention has the potential to meet unmet psychological needs and also global priorities to support people with dementia to 'live well' with dementia and promote 'healthy ageing'. The intervention has further potential to reduce informal caregiver burden by providing effective strategies for informal caregivers to support people with dementia and depression.[31] The planned study is designed to improve the implementation potential of the intervention by utilising NPT. Given barriers to the implementation of evidence-based healthcare interventions are common, NPT will provide a framework for understanding potential barriers and facilitators to implementation from the intervention development phase. This will enhance future implementation potential should the intervention be demonstrated to be clinically and cost effective in the future. Our study design also allows us to collect in-depth qualitative data to provide a rich understanding of how to adapt the intervention from the perspective of people with dementia and informal caregivers. Our careful exploration of the perceived relevance of the intervention content and language and ways to enhance relevancy, cultural appropriateness, and acceptability, will facilitate the development of a more acceptable and relevant intervention, specifically tailored to needs and preferences of the population.

Despite the aforementioned study strengths, there are some limitations. First, people with dementia do not need to have past or present depression to participate. As such, we may recruit people with dementia who have no experience of depression or low mood, thus limiting the transferability of findings to people with dementia and depression. However, we will ask people with dementia about their past and present wellbeing, providing us with some indication as to the experience of depression in the study sample. Second, due to resource limitations, we are unable to include ethnic minority groups who do not speak Swedish due to the increasing costs for e.g., recruitment, informed consent, and need for translated materials. This will limit the transferability of results and sample representativeness, e.g., for people with dementia and a migration background, who are at increased risk of marginalisation and unfortunately there is currently a lack of culturally appropriate interventions for this

For peer review only

population.[84] However, the innovative intervention development process adopted in this study has potential for wider applications and can serve as a template for future adaptations of the intervention for people with dementia and other populations not represented in the present study.

In conclusion, this study will result in the development of a tailored intervention, hopefully optimised to improve relevancy and acceptability for a currently neglected population. Results will be used to inform the design of a phase II feasibility following the MRC framework for the development and evaluation of complex interventions[35] to further explore the feasibility and acceptability of the intervention.

## ETHICS AND DISSEMINATION

The study will be conducted in accordance with Helsinki Declaration.[85] All data will be handled according to General Data Protection Regulation (GDPR).[86] Informed consent will be obtained from all study participants[87] and capacity to consent in people with dementia will be established in accordance with Swedish Health and Medical Services Act.[64] Capacity to consent will be examined by a member from the research team. Given only people with dementia with capacity to consent will be included in this study, we anticipate people with mild to moderate dementia will participate. The study is approved by the Swedish Ethical Review Authority (Dnr: 2020-05542 and Dnr: 2021-00925). Findings will be published in an open access peer-reviewed journal. We will aim to present results at scientific conferences as well as publications for public and professional audiences. The research team and PPI group will also promote results via social media.

## AUTHORS' CONTRIBUTIONS

FS: methodology, resources, writing – original draft, project administration; AB: writing – original draft, project administration, supervision; PF: conceptualisation, writing – review and editing; ACÅ: methodology, writing – review and editing; OB: writing – review and editing, project administration; CC: writing – review and editing, LvE: Writing – review and editing; JW: conceptualisation, methodology, writing - original draft, supervision, project administration, funding acquisition.

## FUNDING

For peer review only

This work is funded by the Swedish Research Council (Dnr: 2018-02691). Author CC's time is funded by the European Union's Horizon 2020 research and innovation programme under the Marie-Skłodowska Curie grant agreement no 814 072. Funders were not involved in the creation, development, or publication of this protocol. Funders will not be involved in the conduct, analysis or reporting of the resulting study.

## COMPETING INTERESTS

The authors declare they have no conflict of interests.

## COPYRIGHT AND AUTHORS' RIGHTS

I, the Submitting Author has the right to grant and does grant on behalf of all authors of the Work (as defined in the below author licence), an exclusive licence and/or a non-exclusive licence for contributions from authors who are: i) UK Crown employees; ii) where BMJ has agreed a CC-BY licence shall apply, and/or iii) in accordance with the terms applicable for US Federal Government officers or employees acting as part of their official duties; on a worldwide, perpetual, irrevocable, royalty-free basis to BMJ Publishing Group Ltd ("BMJ") its licensees and where the relevant Journal is co-owned by BMJ to the co-owners of the Journal, to publish the Work in BMJ Open and any other BMJ products and to exploit all rights, as set out in our licence.

## ABBREVIATIONS

BA: Behavioural Activation

CBT: Cognitive Behavioural Therapy

GRIPP2: Guidance for Reporting Patient and Public Involvement 2

LI-CBT: Low-Intensity Cognitive Behavioural Therapy

MRC: Medical Research Council

NPT: Normalisation Process Theory

PAR: Participatory Action Research

PPI: Patient and Public Involvement

S-NoMAD: Swedish version of the Normalisation Process Theory Measure

SRQR: Standards for Reporting Qualitative Research

TIDieR: Template for Intervention Description and Replication

## FIGURES

For peer review only

Figure 1. Overall model of delivery of the Low-Intensity BA intervention developed in England.

Figure 2. An overview of the data collection procedure and topics for the four PAR Phases.

For peer review only

## REFERENCES

1 Nichols E, Szoeke CE, Vollset SE, et al. Global, regional, and national burden of Alzheimer's disease and other dementias, 1990–2016: a systematic analysis for the Global Burden of Disease Study 2016. *Lancet Neurol* 2019;18:88-106. doi:10.1016/S1474-4422(18)30403-4.

2 Livingston G, Sommerlad A, Orgeta V, et al. Dementia prevention, intervention, and care. *Lancet* 2017;390:2673-734. doi:10.1016/S0140-6736(17)31363-6.

3 Wimo A, Jönsson L, Fratiglioni L, et al. The societal costs of dementia in Sweden 2012 – relevance and methodological challenges in valuing informal care. *Alzheimers Res Ther* 2016;8:59. doi:10.1186/s13195-016-0215-9.

4 Wright T, O'Connor S. Reviewing challenges and gaps in European and global dementia policy. *J Public Ment Health* 2018;17:157-67. doi:10.1108/JPMH-02-2018-0012.

5 Ritchie CW, Terrera GM, Quinn TJ. Dementia trials and dementia tribulations: methodological and analytical challenges in dementia research. *Alzheimers Res Ther* 2015;7:31. doi:10.1186/s13195-015-0113-6.

6 Sleeman KE, de Brito M, Etkind S, et al. The escalating global burden of serious health-related suffering: projections to 2060 by world regions, age groups, and health conditions. *Lancet Glob Health* 2019;7:e883-92. doi:10.1016/S2214-109X(19)30172-X.

7 Prince M, Acosta D, Ferri CP, et al. Dementia incidence and mortality in middle-income countries, and associations with indicators of cognitive reserve: a 10/66 dementia research group population-based cohort study. *Lancet* 2012;380:50-8. doi:10.1016/S0140-6736(12)60399-7.

8 Holopainen A, Siltanen H, Pohjanvuori A, et al. Factors associated with the quality of life of people with dementia and with quality of life-improving interventions: scoping review. *Dementia (London)* 2019;18:1507-37. doi:10.1177/1471301217716725.

9 Laver K, Dyer S, Whitehead C, et al. Interventions to delay functional decline in people with dementia: a systematic review of systematic reviews. *BMJ Open* 2016;6:e010767. doi:10.1136/bmjopen-2015-010767.

10 Cipriani G, Lucetti C, Carlesi C, et al. Depression and dementia. A review. *Eur Geriatr Med* 2015;6:479-86. doi:10.1016/j.eurger.2015.07.010.

11 Enache D, Winblad, Aarsland D. Depression in dementia: epidemiology, mechanisms, and treatment. *Curr Opin Psychiatry* 2011;24:461-72.

For peer review only

doi:10.1097/YCO.0b013e32834bb9d4.
12 Modrego PJ. Depression in Alzheimer's Disease. Pathophysiology, diagnosis, and treatment. *J Alzheimers Dis* 2010;21:1077-87. doi:10.3233/JAD-2010-100153.
13 Steinberg M, Shao H, Zandi P, et al. Point and 5-year period prevalence of neuropsychiatric symptoms in dementia: the Cache County Study. *Int J of Geriatric Psychiatry* 2008;23:170-7. doi:10.1002/gps.1858.
14 Curran EM, Loi S. Depression and dementia. *Med J Aust* 2013;199:40-4. doi:10.5694/mjao12.10567.
15 Kitching D. Depression in dementia. *Aust prescr* 2015;38:209-11. doi:10.18773/austprescr.2015.071.
16 Orgeta V, Qazi A, Spector AE, et al. Psychological treatments for depression and anxiety in dementia and mild cognitive impairment. *Cochrane Database Syst Rev* 2014;1:CD009125. doi:10.1002/14651858.CD009125.pub2.
17 van der Roest HG, Meiland FJM, Comijs HC, et al. What do community-dwelling people with dementia need? A survey of those who are known to care and welfare services. *Int Psychogeriatr* 2009;21:949-65. doi:10.1017/S1041610209990147.
18 Kishita N, Backhouse T, Mioshi E. Nonpharmacological interventions to improve depression, anxiety, and quality of life (QoL) in people with dementia: an overview of systematic reviews. *J Geriatr Psychiatry Neurol* 2019;33:28-41. doi:10.1177/0891988719856690.
19 Leyhe T, Reynolds III CF, Melcher T, et al. A common challenge in older adults: classification, overlap, and therapy of depression and dementia. *Alzheimers Dement* 2017;13:59-71. doi:10.1016/j.jalz.2016.08.007.
20 Wittchen HU, Jacobi F, Rehm J, et al. The size and burden of mental disorders and other disorders of the brain in Europe 2010. *Eur Neuropsychopharmacol* 2011;21:655-79. doi:10.1016/J.EURONEURO.2011.07.018.
21 Bennett-Levy J, Richards DA, Farrand P. Low intensity CBT interventions: a revolution in mental health care. In: Bennett-Levy J, eds. Oxford guide to low intensity CBT interventions. Oxford: Oxford University Press 2010;3-18.
22 Rebello TJ, Marques A, Gureje O, et al. Innovative strategies for closing the mental health treatment gap globally. *Curr Opin Psychiatry* 2014;27:308-14. doi:10.1097/YCO.0000000000000068.
23 Farrand P. Low-intensity CBT skills and interventions: a practitioners′ manual. London: SAGE Publications Ltd 2020.

For peer review only

24 Roth A, Pilling S. The competencies required to deliver effective cognitive and behavioural therapy for people with depression and anxiety disorders. London: Department of Health 2007.

25 Farrand P, Pentecost C, Greaves C, et al. A written self-help intervention for depressed adults comparing behavioural activation combined with physical activity promotion with a self-help intervention based upon behavioural activation alone: study protocol for a parallel group pilot randomised controlled trial (BAcPAc). *Trials* 2014;15:196. doi:10.1186/1745-6215-15-196.

26 Leventhal AM. Sadness, depression, and avoidance behavior. *Behav Modif* 2008;32:759-79. doi:10.1177/0145445508317167.

27 Kerkhof Y, Pelgrum-Keurhorst M, Mangiaracina F, et al. User-participatory development of FindMyApps; a tool to help people with mild dementia find supportive apps for self-management and meaningful activities. *Digit Health* 2019;5:1-19. doi:10.1177/2055207618822942.

28 Copeland JL, Ashe MC, Biddle SJ, et al. Sedentary time in older adults: a critical review of measurement, associations with health, and interventions. *Br J Sports Med* 2017;51:1539. doi:10.1136/bjsports-2016-097210.

29 Farrand P, Woodford J, Small F, et al. Behavioural activation self-help to improve depression in people living with dementia: the PROMOTE treatment protocol. *NZ J Psychol* 2017;46:51.

30 Dodel R, Belger M, Reed C, et al. Determinants of societal costs in Alzheimer's disease: GERAS study baseline results. *Alzheimers Dement* 2015;11:933-45. doi:10.1016/J.JALZ.2015.02.005.

31 Mausbach BT, Patterson TL, Grant I. Is depression in Alzheimer's caregivers really due to activity restriction? A preliminary mediational test of the Activity Restriction Model. *J Behav Ther Exp Psychiatry* 2008;39:459-66. doi:10.1016/J.JBTEP.2007.12.001.

32 Prince M, Bryce R, Albanese E, et al. The global prevalence of dementia: a systematic review and metaanalysis. *Alzheimers Dement* 2013;9:63-75.e2. doi:10.1016/J.JALZ.2012.11.007.

33 Kasper JD, Freedman VA, Spillman BC, et al. The disproportionate impact of dementia on family and unpaid caregiving to older adults. *Health Aff* 2015;34:1642-9. doi:10.1377/hlthaff.2015.0536.

34 Bleijenberg N, de Man-van Ginkel, Janneke M, et al. Increasing value and reducing

For peer review only

waste by optimizing the development of complex interventions: enriching the development phase of the Medical Research Council (MRC) Framework. *Int J Nurs Stud* 2018;79:86-93. doi:10.1016/j.ijnurstu.2017.12.001.

35 Craig P, Dieppe P, Macintyre S, et al. Developing and evaluating complex interventions: the new Medical Research Council guidance. *BMJ* 2008;337:a1655. doi:10.1136/bmj.a1655.

36 Heim E, Kohrt BA. Cultural adaptation of scalable psychological interventions: a new conceptual framework. *Clin Psychol Eur* 2019;1:1-22. doi:10.32872/cpe.v1i4.37679.

37 Castro FG, Barrera M, Holleran Steiker LK. Issues and challenges in the design of culturally adapted evidence-based interventions. *Annu Rev Clin Psychol* 2010;6:213-39. doi:10.1146/annurev-clinpsy-033109-132032.

38 Ferrer-Wreder L, Adamson L, Kumpfer KL, et al. Advancing intervention science through effectiveness research: a global perspective. *Child Youth Care Forum* 2012;41:109-17. doi:10.1007/s10566-012-9173-y.

39 Bernal G, Jiménez-Chafey MI, Domenech Rodríguez MM. Cultural adaptation of treatments: a resource for considering culture in evidence-based practice. *Prof Psychol Res Pr* 2009;40:361-8. doi:10.1037/a0016401.

40 Grundström H, Rauden A, Olovsson M. Cross-cultural adaptation of the Swedish version of Endometriosis Health Profile-30. *J Obstet Gynaecol* 2020;40:969-73. doi:10.1080/01443615.2019.1676215.

41 Evans RE, Craig P, Hoddinott P, et al. When and how do 'effective' interventions need to be adapted and/or re-evaluated in new contexts? The need for guidance. *J Epidemiol Community Health* 2019;73:481-2. doi:10.1136/jech-2018-210840.

42 Pfadenhauer LM, Gerhardus A, Mozygemba K, et al. Making sense of complexity in context and implementation: the Context and Implementation of Complex Interventions (CICI) framework. *Implement Sci* 2017;12:21. doi:10.1186/s13012-017-0552-5.

43 Brantnell A, Woodford J, Baraldi E, et al. Views of implementers and nonimplementers of internet-administered cognitive behavioral therapy for depression and anxiety: survey of primary care decision makers in Sweden. *J Med Internet Res* 2020;22:e18033. doi:10.2196/18033.

44 Bieber A, Stephan A, Verbeek H, et al. Access to community care for people with dementia and their informal carers: case vignettes for a European comparison of structures and common pathways to formal care. *Z Gerontol Geriatr* 2018;51:530-6.

For peer review only

doi:10.1007/s00391-017-1266-7.

45 Bach-Mortensen AM, Lange BCL, Montgomery P. Barriers and facilitators to implementing evidence-based interventions among third sector organisations: a systematic review. *Implement Sci* 2018;13:103-19. doi:10.1186/s13012-018-0789-7.

46 Murray E, Treweek S, Pope C, et al. Normalisation process theory: a framework for developing, evaluating and implementing complex interventions. *BMC Med* 2010;8:63. doi:10.1186/1741-7015-8-63.

47 May C, Finch T. Implementing, embedding, and integrating practices: an outline of normalization process theory. *Sociology* 2009;43:535-54. doi:10.1177/0038038509103208.

48 May CR, Cummings A, Girling M, et al. Using normalization process theory in feasibility studies and process evaluations of complex healthcare interventions: a systematic review. *Implement Sci* 2018;13:80. doi:10.1186/s13012-018-0758-1.

49 May C, Rapley T, Mair FS, et al. Normalization process theory on-line users' manual, toolkit and NoMAD instrument. 2015. Available from: http://www.normalizationprocess.org. [Accessed 2021 May 27].

50 McEvoy R, Ballini L, Maltoni S, et al. A qualitative systematic review of studies using the normalization process theory to research implementation processes. *Implement Sci* 2014;9:2. doi:10.1186/1748-5908-9-2.

51 Mills K, Griffin SJ, Sutton S, et al. Development and usability testing of a very brief intervention for personalised cancer risk assessment to promote behaviour change in primary care using normalisation process theory. *Prim Health Care Res Dev* 2020;21:e1. doi:10.1017/S146342361900080X.

52 Early F, Wilson P, Deaton C, et al. Developing an intervention to increase REferral and uptake TO pulmonary REhabilitation in primary care in patients with chronic obstructive pulmonary disease (the REsTORE study): mixed methods study protocol. *BMJ Open* 2019;9:e024806. doi:10.1136/bmjopen-2018-024806.

53 Goeman D, Michael J, King J, et al. Partnering with consumers to develop and evaluate a Vietnamese Dementia Talking-Book to support low health literacy: a qualitative study incorporating. *BMJ Open* 2016;6:e011451. doi:10.1136/bmjopen-2016-011451.

54 Sandlund M, Lindgren H, Pohl P, et al. Towards a mobile exercise application to prevent falls: a participatory design process. *Int J Child Health Hum Dev* 2016;9:389-98.

For peer review only

55 Nygren M, Roback K, Öhrn A, et al. Factors influencing patient safety in Sweden: perceptions of patient safety officers in the county councils. *BMC Health Serv Res* 2013;13:52. doi:10.1186/1472-6963-13-52.

56 Socialtjänstlag (SFS 2001:453). Stockholm: Socialdepartementet. Available from: https://www.riksdagen.se/sv/dokument-lagar/dokument/svensk-forfattningssamling/socialtjanstlag-2001453_sfs-2001-453. [Accessed 2021 May 27].

57 Statistiska centralbyrån. Folkmängd i riket, län och kommuner 31 mars 2021 och befolkningsförändringar 1 januari - 31 mars 2021. Totalt. Stockholm: Statistiska centralbyrån; 2019. Available from: https://www.scb.se/hitta-statistik/statistik-efter-amne/befolkning/befolkningens-sammansattning/befolkningsstatistik/pong/tabell-och-diagram/kvartals--och-halvarsstatistik--kommun-lan-och-riket/folkmangd-i-riket-lan-och-kommuner-31-mars-2021-och-befolkningsforandringar-1-januari---31-mars-2021.-totalt/. [Accessed 2021 May 27].

58 Region Uppsala. Demenssjukdomar - kliniska riktlinjer. Uppsala: Region Uppsala; 2018. Available from: http://publikdocplus.regionuppsala.se/Home/GetDocument?containerName=e0c73411-be4b-4fee-ac09-640f9e2c5d83&reference=DocPlusSTYR-4202&docId=DocPlusSTYR-4202&filename=Demenssjukdomar%20-%20kliniska%20riktlinjer.pdf. [Accessed 2021 May 27].

59 Hegde S, Ellajosyula R. Capacity issues and decision-making in dementia. *Ann Indian Acad Neurol* 2016;19(Suppl 1):S34-S39. doi:10.4103/0972-2327.192890.

60 Saunders B, Sim J, Kingstone T, et al. Saturation in qualitative research: exploring its conceptualization and operationalization. *Qual Quant* 2018;52:1893-907. doi:10.1007/s11135-017-0574-8.

61 Sim J, Saunders B, Waterfield J, et al. Can sample size in qualitative research be determined a priori? *Int J Soc Res Methodol* 2018;21:619-34. doi:10.1080/13645579.2018.1454643.

62 Rabiee F. Focus-group interview and data analysis. *Proc Nutr Soc* 2004;63:655-60. doi:10.1079/pns2004399.

63 O'Connor S, Hanlon P, O'Donnell CA, et al. Understanding factors affecting patient and public engagement and recruitment to digital health interventions: a systematic review of qualitative studies. *BMC Med Inform Decis Mak* 2016;1:120. doi:10.1186/s12911-016-0359-3.

64 Hälso- och sjukvårdslag (SFS 2017:30). Stockholm: Socialdepartementet. Available

For peer review only

from: https://www.riksdagen.se/sv/dokument-lagar/dokument/svensk-forfattningssamling/halso--och-sjukvardslag_sfs-2017-30. [Accessed 2021 May 27].

65 Warner J, McCarney R, Griffin M, et al. Participation in dementia research: rates and correlates of capacity to give informed consent. *J Med Ethics* 2008;34:167-70. doi:10.1136/jme.2006.019786.

66 Dementia Engagement and Empowerment Project. Writing dementia-friendly information. Swindon: Dementia Engagement and Empowerment Project; 2013. Available from: https://www.dementiavoices.org.uk/wp-content/uploads/2013/11/DEEP-Guide-Writing-dementia-friendly-information.pdf. [Accessed 2021 May 27].

67 Barnes M, Wiles N, Morrison J, et al. Exploring patients' reasons for declining contact in a cognitive behavioural therapy randomised controlled trial in primary care. *Br J Gen Pract* 2012;62:e371-7. doi:10.3399/bjgp12X641492.

68 Farrand P, Woodford J, Llewellyn D, et al. Behavioural activation written self-help to improve mood, wellbeing and quality of life in people with dementia supported by informal carers (PROMOTE): a study protocol for a single-arm feasibility study. *Pilot Feasibility Stud* 2016;2:42. doi:10.1186/s40814-016-0083-x.

69 Woodford J, Farrand P, Watkins ER, et al. Supported cognitive-behavioural self-help versus treatment-as-usual for depressed informal carers of stroke survivors (CEDArS): study protocol for a feasibility randomized controlled trial. *Trials* 2014;15:157. doi:10.1186/1745-6215-15-157.

70 Farrand P, Woodford J, Anderson M, et al. Getting More Out of Every Day with Memory Difficulties: A Guide for People Living with Memory Difficulties. Exeter: University of Exeter 2015a.

71 Farrand P, Woodford J, Anderson M. Getting More Out of Every Day with Memory Difficulties: A Guide for Family and Friends. Exeter: University of Exeter 2015b.

72 Roth AD, Pilling S. Using an evidence-based methodology to identify the competences required to deliver effective cognitive and behavioural therapy for depression and anxiety disorders. *Behav Cogn Psychother* 2008;36:129-47. doi:10.1017/S1352465808004141.

73 Heslop CW, Burns S, Lobo R, et al. Developing a framework for community-based sexual health interventions for youth in the rural setting: protocol for a participatory action research study. *BMJ Open* 2017;7:e013368. doi:10.1136/bmjopen-2016-013368.

For peer review only

74 Elf M, Nordmark S, Lyhagen J, et al. The Swedish version of the Normalization Process Theory Measure S-NoMAD: translation, adaptation, and pilot testing. *Implement Sci* 2018;13:146. doi:10.1186/s13012-018-0835-5.

75 Finch TL, Rapley T, Girling M, et al. Improving the normalization of complex interventions: measure development based on normalization process theory (NoMAD): study protocol. *Implement Sci* 2013;8:43. doi:10.1186/1748-5908-8-43.

76 Elo S, Kyngäs H. The qualitative content analysis process. *J Adv Nurs* 2008;62:107-15. doi:10.1111/j.1365-2648.2007.04569.x.

77 Krefting L. Rigor in qualitative research: the assessment of trustworthiness. *Am J Occup Ther* 1991;45:214-22. doi:10.5014/ajot.45.3.214.

78 Elo S, Kääriäinen M, Kanste O, et al. Qualitative content analysis: a focus on trustworthiness. *SAGE Open* 2014;4:1-10. doi:10.1177/2158244014522633.

79 O'Brien BC, Harris IB, Beckman TJ, et al. Standards for reporting qualitative research: a synthesis of recommendations. *Acad Med* 2014;89:1245-51. doi:10.1097/ACM.0000000000000388.

80 Hoffmann TC, Glasziou PP, Boutron I, et al. Better reporting of interventions: template for intervention description and replication (TIDieR) checklist and guide. *BMJ* 2014;348:g1687. doi:10.1136/bmj.g1687.

81 Staniszewska S, Brett J, Simera I, et al. GRIPP2 reporting checklists: tools to improve reporting of patient and public involvement in research. *BMJ* 2017;358:j3453. doi:10.1136/bmj.j3453.

82 Mann C, Chilcott S, Plumb K, et al. Reporting and appraising the context, process and impact of PPI on contributors, researchers and the trial during a randomised controlled trial - the 3D study. *Res Involv Engagem* 2018;4:15. doi:10.1186/s40900-018-0098-y.

83 Socialstyrelsen. Nationella riktlinjer för vård och omsorg vid demenssjukdom: stöd för styrning och ledning. Stockholm: Socialstyrelsen; 2017. Available from: https://www.socialstyrelsen.se/globalassets/sharepoint-dokument/artikelkatalog/nationella-riktlinjer/2017-12-2.pdf. [Accessed 2021 May 27].

84 Nielsen TR, Nielsen DS, Waldemar G. Barriers in access to dementia care in minority ethnic groups in Denmark: a qualitative study. *Aging Ment Health* 2020;1-9. doi:10.1080/13607863.2020.1787336.

85 World Medical Association. World medical association declaration of helsinki: ehical principles for medical research involving human subjects. *JAMA* 2013;310:2191-4. doi:10.1001/jama.2013.281053.

For peer review only

86 European Parliament and the Council of the European Union. Regulation (EU) 2016/679 of the European Parliament and of the Council of 27 April 2016 on the protection of natural persons with regard to the processing of personal data and on the free movement of such data, and repealing Directive 95/46. *OJEU* 2016;59:1-88.

87 Lag om etikprövning av forskning som avser människor (SFS 2004:460). Stockholm: Utbildningsdepartementet. Available from: https://www.riksdagen.se/sv/dokument-lagar/dokument/svensk-forfattningssamling/lag-2003460-om-etikprovning-av-forskning-som_sfs-2003-460. [Accessed 2021 May 27].

For peer review only

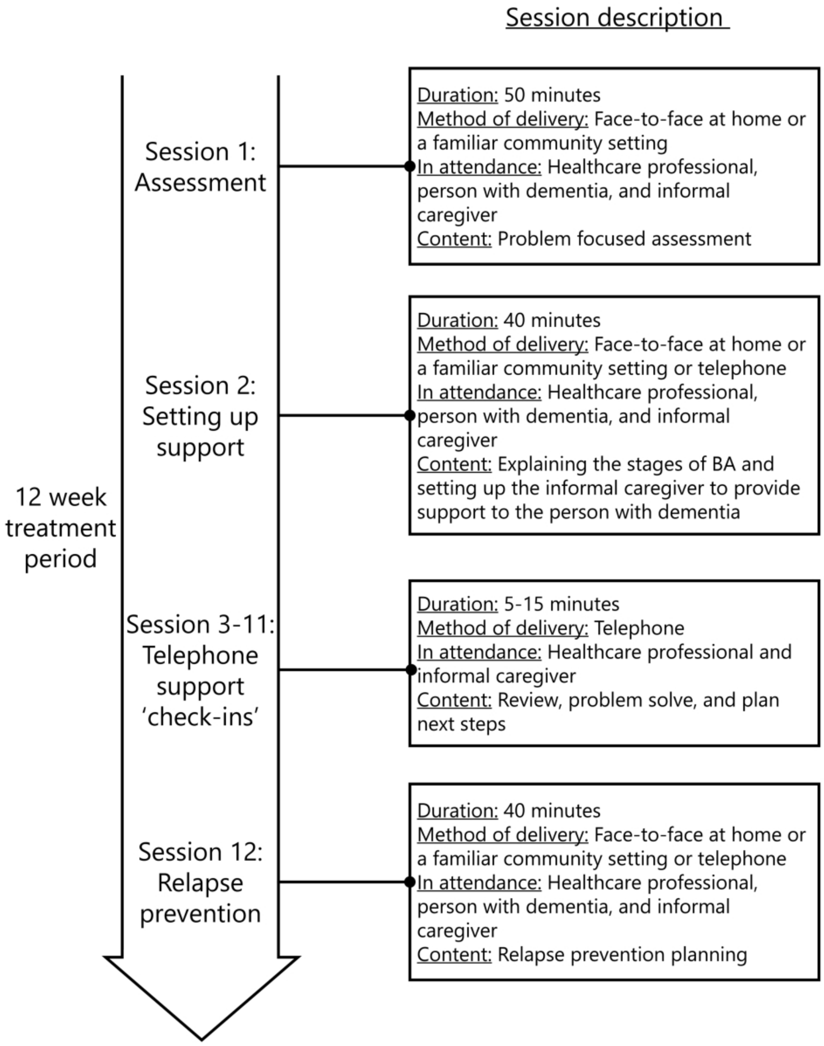


Figure 1. Overall model of delivery of the Low-Intensity BA intervention developed in England.

209x296mm (300 x 300 DPI)

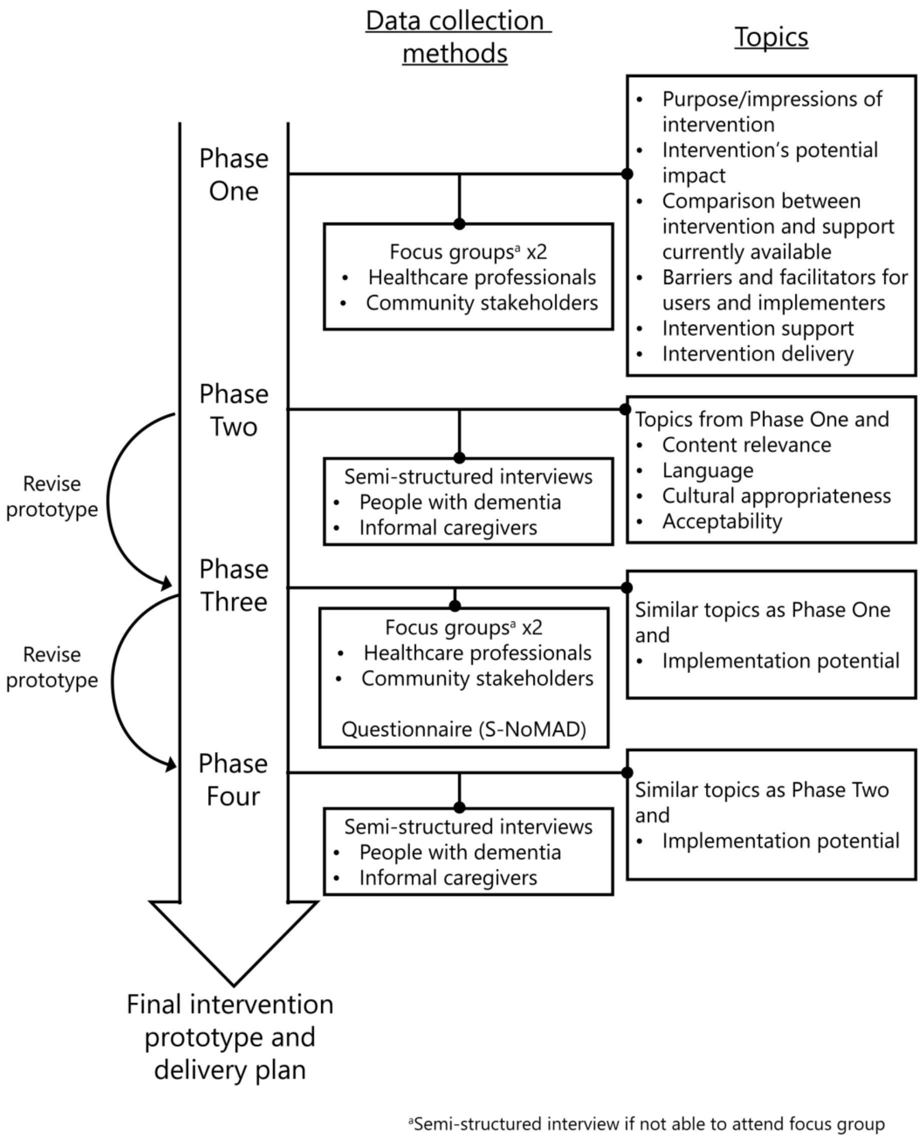


Figure 2. An overview of the data collection procedure and topics for the four PAR Phases.

215x279mm (300 x 300 DPI)
